# Impact of London's road traffic air and noise pollution on birth weight: retrospective population based cohort study

**DOI:** 10.1136/bmj.j5299

**Published:** 2017-12-05

**Authors:** Rachel B Smith, Daniela Fecht, John Gulliver, Sean D Beevers, David Dajnak, Marta Blangiardo, Rebecca E Ghosh, Anna L Hansell, Frank J Kelly, H Ross Anderson, Mireille B Toledano

**Affiliations:** 1 MRC-PHE Centre for Environment and Health, Department of Epidemiology and Biostatistics, School of Public Health, Imperial College London, St Mary’s Campus, Norfolk Place, London W2 1PG, UK; 2 NIHR HPRU in Health Impact of Environmental Hazards, King's College London, London, UK; 3 UK Small Area Health Statistics Unit, MRC-PHE Centre for Environment and Health, Department of Epidemiology and Biostatistics, School of Public Health, Imperial College London, London, UK; 4 MRC-PHE Centre for Environment and Health, Environmental Research Group, Faculty of Life Sciences and Medicine, King's College London, London, UK; 5 Population Health Research Institute, St George’s, University of London, London, UK

## Abstract

**Objective:**

To investigate the relation between exposure to both air and noise pollution from road traffic and birth weight outcomes.

**Design:**

Retrospective population based cohort study.

**Setting:**

Greater London and surrounding counties up to the M25 motorway (2317 km^2^), UK, from 2006 to 2010.

**Participants:**

540 365 singleton term live births.

**Main outcome measures:**

Term low birth weight (LBW), small for gestational age (SGA) at term, and term birth weight.

**Results:**

Average air pollutant exposures across pregnancy were 41 μg/m^3^ nitrogen dioxide (NO_2_), 73 μg/m^3^ nitrogen oxides (NO_x_), 14 μg/m^3^ particulate matter with aerodynamic diameter <2.5 μm (PM_2.5_), 23 μg/m^3^ particulate matter with aerodynamic diameter <10 μm (PM_10_), and 32 μg/m^3^ ozone (O_3_). Average daytime (L_Aeq,16hr_) and night-time (L_night_) road traffic A-weighted noise levels were 58 dB and 53 dB respectively. Interquartile range increases in NO_2_, NO_x_, PM_2.5_, PM_10_, and source specific PM_2.5_ from traffic exhaust (PM_2.5 traffic exhaust_) and traffic non-exhaust (brake or tyre wear and resuspension) (PM_2.5 traffic non-exhaust_) were associated with 2% to 6% increased odds of term LBW, and 1% to 3% increased odds of term SGA. Air pollutant associations were robust to adjustment for road traffic noise. Trends of decreasing birth weight across increasing road traffic noise categories were observed, but were strongly attenuated when adjusted for primary traffic related air pollutants. Only PM_2.5 traffic exhaust_ and PM_2.5_ were consistently associated with increased risk of term LBW after adjustment for each of the other air pollutants. It was estimated that 3% of term LBW cases in London are directly attributable to residential exposure to PM_2.5_>13.8 μg/m^3^during pregnancy.

**Conclusions:**

The findings suggest that air pollution from road traffic in London is adversely affecting fetal growth. The results suggest little evidence for an independent exposure-response effect of traffic related noise on birth weight outcomes.

## Introduction

Air pollution is a major public health issue. It has been associated with reduced fetal growth,^1^ through which it may have extensive and permanent influences on the life course.^2^ A key contributor to urban ambient pollution is road traffic and, critically, vehicle emissions are released near people. Urban particulate matter includes a large contribution from outside the urban area, and locally emitted particles. Close to roads an individual would be exposed to more primary exhaust and non-exhaust (brake or tyre wear and resuspension of road dust induced by vehicles) particles. Further away from roads an individual would be exposed to more nitrate and secondary organic aerosol as a proportion of their total particulate dose.

Road traffic also produces noise, which has been associated with adverse health outcomes such as hypertension and cardiovascular disease.^3^ Research on how noise affects birth outcomes is more limited, but a possible effect on LBW has been suggested.^4^ Noise could potentially influence fetal growth through stress, hypertension, and sleep disturbance.^4^ ^5^ ^6^

Evidence about the relative roles of air and noise pollution on birth weight is limited and inconsistent.^7^ ^8^ ^9^ To address health impacts of traffic effectively these need to be better understood. In this study, we investigate long term exposure to both traffic related air and noise pollution during pregnancy in relation to birth weight outcomes.

## Methods

### Births data

**Fig 1:**
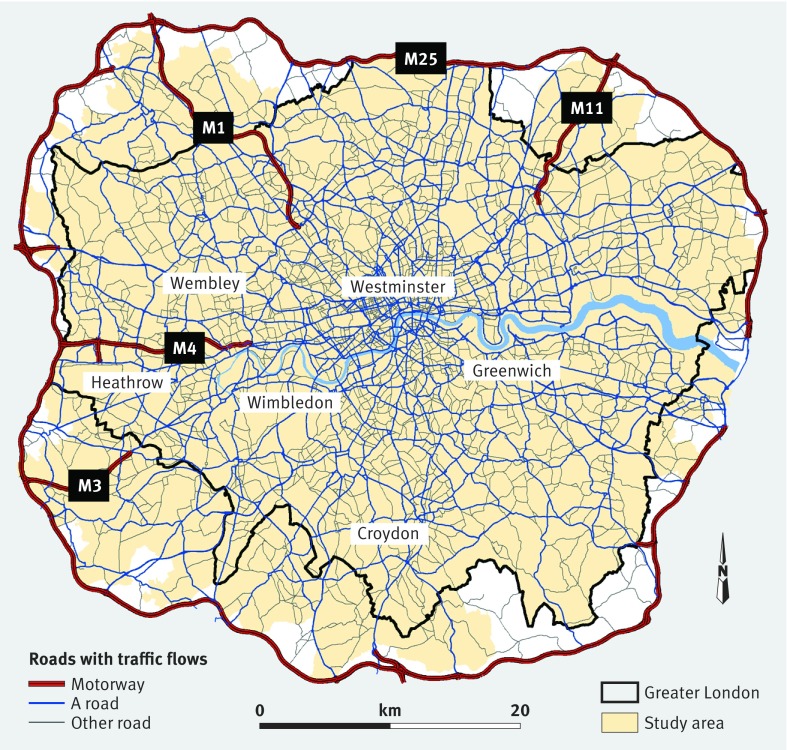
Map of study area

The study boundary was the M25, an orbital motorway encompassing all of Greater London and parts of other counties (2317 km^2^), as traffic information, and therefore air pollution and noise estimates, was not available for beyond the M25. Figure 1 shows the study area. We extracted 671 509 singleton births occurring within the M25 from 2006 to 2010 from the UK National Births and Stillbirth registers held at the UK Small Area Health Statistics Unit and supplied by the Office for National Statistics. These registers provide routinely collected data on all births in the country, including date of birth, birth weight, sex, and mother’s age. We appended gestational age and baby’s ethnicity from the NHS Numbers for Babies (NN4B) dataset, with 99.2% linkage. The method of gestational age assessment is not recorded on NN4B records. It is likely to be based on the more accurate and recent information from a mother’s routine second trimester scan but a proportion may be based on the date of the last menstrual period.^10^

Maternal residential addresses at the time of birth were geocoded to 0.1 m accuracy using Quick Address Software (Experian, 2015). We did not have information on whether a mother changed address during pregnancy. We excluded births in middle layer super output areas overlapping the M25 (n=7493) because area level covariates would reflect populations inside and outside the study boundary. We obtained 2011 census output area level data as follows: Carstairs deprivation index from UK Census 2011 standardised across census output areas in study area;^11^ and 2014 tobacco expenditure each week (population ≥16 years) from CACI, as a smoking proxy.

### Air pollution exposures

Average monthly concentrations of nitrogen dioxide (NO_2_), nitrogen oxides (NO_x_), ozone (O_3_), particulate matter with diameter <2.5 μm (PM_2.5_), particulate matter with diameter <10 μm (PM_10_), PM_2.5_ from traffic exhaust (PM_2.5 traffic exhaust_), and PM_2.5_ from traffic non-exhaust (PM_2.5 traffic non-exhaust_) were estimated for points on a 20 m × 20 m regular grid across the study area, using dispersion modelling (KCLurban).^12^ NO_2_, NO_x_, PM_2.5 traffic exhaust_, and PM_2.5 traffic non-exhaust_ are primary pollutants related to traffic (ie, locally emitted or rapidly formed near source oxidation products, or both). PM_2.5_ and PM_10_ are dominated by regional particles, long range particles, and secondary particles formed through atmospheric chemical reactions but also include particles from primary traffic sources. O_3_ is a regional, secondary pollutant. PM_2.5_, PM_10_, and O_3_ are more homogeneously distributed than primary pollutants related to traffic. 

The KCLurban model uses Atmospheric Dispersion Modelling System (version 4) and road source model (version 2.3); data on emissions from the London Atmospheric Emissions Inventory (LAEI);^13^ empirically derived NO-NO_2_-O_3_ and PM relations; and hourly meteorological information.^12^ The model performed well when evaluated against measurements, with high spearman correlation coefficients (ρ) between observed versus modelled monthly concentrations: ρ>0.91 for NO_x_, PM_10_, and PM_2.5_; ρ>0.83 for NO_2_; and ρ>0.9 for O_3_ at both roadside and background locations.^14^ Normalised mean bias (NMB) and root mean square error (RMSE) for modelled monthly predictions were slightly higher for NO_x_ (NMB 11%; RMSE 13 μg/m^3^, 22%) and NO_2_ (11%; 5.2 μg/m^3^, 20%) compared with PM_2.5_ (5%; 2.2 μg/m^3^, 14%) and PM_10_ ( 6%; 3.1 μg/m^3^, 12%), indicating that whilst all have a positive bias (NMB), PM_2.5_ and PM_10_ are more accurately predicted than NO_2_ and NO_x_ (RMSE). Further detail about the modelling procedure and model evaluation is available elsewhere.^12^ ^14^ Using a Geographic Information System, each maternal residential address was assigned monthly air pollutant concentrations for the nearest 20 m × 20 m grid point according to its geocoded XY coordinates. For each birth record, we calculated the time weighted average concentrations for NO_x_, NO_2_, PM_2.5 traffic exhaust_, PM_2.5 traffic non-exhaust_, PM_2.5_, PM_10_, and O_3_ across pregnancy and for each trimester (first trimester defined as days 1-93, second as days 94-186, and third as day 187 to day preceding delivery). The time weighting was based on the proportion of the pregnancy or trimester in each calendar month.^15^ ^16^ To define trimesters, gestation period (available as completed weeks of pregnancy) was converted to days, and 4 days (rounded up from the midpoint 3.5 days) was added to adjust for potential underestimation where true gestation period was not an exact number of completed weeks.

### Road traffic noise exposures

A-weighted road traffic noise levels (dB) were modelled to 0.1 dB resolution for all geocoded maternal residential addresses using the Traffic Noise Exposure (TRANEX) model:^17^ L_Aeq,16hr_ (average sound level 0700-2300 hours); L_night_ (2300-0700); L_day_ (0700-1900); L_eve_ (1900-2300); L_den_ (logarithmic composite of L_day_, L_eve_, and L_night_ with 5 dB added to the L_eve_ and 10 dB added to L_night_). Model validation studies conducted in two UK cities showed high Spearman’s correlation (ρ=0.90) between measured and modelled noise levels, indicating good model performance.^17^ The geocoded address points are for the geometric centroid of the dwelling, so for the purposes of noise modelling, the address points were universally moved to one metre from the façade on the side of the dwelling closest to the nearest road section with traffic information, as described elsewhere.^17^ We modelled noise for one midpoint year (2007) and applied these values to other years for the same address locations because temporal variability in noise over the study period was negligible. Noise could not be estimated for 4.5% of births owing to maternal residential address point (receptor) placement issues,^17^ however, these addresses were randomly distributed across the study area. We flagged addresses exposed to A-weighted L_day_>50 dB from railways or aircraft (Heathrow Airport and London City Airport). Railway and London City Airport noise data were from Environmental Noise Directive strategic noise mapping (2006 annual average), and Heathrow Airport noise data were from annual average contours (2001) from the Civil Aviation Authority.

### Outcomes

Term low birth weight (LBW) was defined as birth weight less than 2500 g and gestational age of 37 weeks or more.^18^ SGA was defined as birth weight for gestational age less than the 10th centile by sex and ethnicity (to account for constitutional differences in birth weight by sex and ethnic group, and thus better identify pathologically small infants). 

We initially excluded births with gestational age less than 24 or greater than 44 weeks (n=1083, 0.2%), missing or implausible (<200 g or >9000 g) birth weight (n=5747, 0.9%), and missing gestational age (n=9725, 1.5%). Birth weight outliers were then identified and excluded according to Tukey’s rule (ie, values greater than twice the interquartile range (IQR), below the first quartile, and above the third quartile for each gestational week) both overall and separately according to sex and ethnicity (white, Asian, black, or other) for the calculation of sex-ethnicity specific birth weight for gestational age centiles.^19^ Stillbirths were retained at this stage, because excluding stillbirths overestimates centiles for gestation <28 weeks by up to 30%.^20^ We identified 0.58% of the observations overall as outliers. We calculated smoothed sex-ethnicity specific birth weight for gestational age centile curves according to the LMS method using LMSChartMaker Light V.2.54 software which has been used in previous research.^21^ ^22^ ^23^ ^24^ ^25^ ^26^ The software can hold a maximum of 100 000 records, so a subsample of 100 000 was randomly selected if the number of records for a given sex and ethnicity subgroup exceeded this. Representativeness of these 100 000 samples for their particular subgroup with respect to exposures or potential confounders was checked and confirmed. We did not calculate centiles or SGA for the ethnic group ‘other’, as it does not represent a meaningful homogeneous ethnic group for analysis. 

We excluded birth weight outliers (n=3815, 0.6%), stillbirths (3910, 0.6%), preterm births (40 346, 6.1%), births missing noise exposure (31 197, 4.7%), and births missing ethnicity (47 710, 7.2%), leaving 540 365 singleton term live births eligible for birth weight analyses, and 471 489 for SGA analyses (the exclusions were not mutually exclusive). 

### Statistical Methods

Air pollutant exposures were analysed as continuous measures, rescaled to both IQR increments and increments specific to pollutants (NO_2_, 10 μg/m^3^; NO_x_, 20 μg/m^3^; PM_2.5 traffic exhaust_, 1 μg/m^3^; PM_2.5 traffic non-exhaust_, 1 μg/m^3^; PM_2.5_, 5 μg/m^3^; PM_10_, 10 μg/m^3^; O_3_, 10 μg/m^3^). Where multiple air pollutants are examined it is a common approach to rescale to the IQR, in order to calculate effect estimates for comparable increases across the different pollutants (which may have very different absolute concentration ranges). The IQR is the difference between the 75th and 25th centiles of the distribution. As all noise metrics were highly correlated (ρ≥0.997), we limited analysis to one daytime (L_Aeq,16hr_) and one night-time (L_night_) metric. Noise metrics were right skewed, so were categorised (L_Aeq,16hr_ <55 dB (reference), 55 to <60 dB, 60 to <65 dB, and ≥65 dB; and L_night_ <50 dB (reference), 50 to <55 dB, 55 to <60 dB, 60 to <65 dB, and ≥65 dB) for primary analysis. We examined the functional relation between term birth weight and noise (supplementary figure 1 in web appendix 1) using generalised additive models, and there were no major departures from linearity so we additionally analysed noise as a continuous variable, rescaled to IQR increment.

We analysed continuous birth weight using linear regression, and LBW or SGA using logistic regression. We limited analyses to term births. We adjusted all models for maternal age (<25, 25-29, 30-34, or ≥35 years); birth registration type (within marriage, sole registration, joint with same address, joint with different address); birth season; birth year; Carstairs deprivation quintile; tobacco expenditure (continuous); and a random intercept for middle layer super output areas. Birth weight and LBW were also adjusted for sex, gestational age (linear and quadratic terms), and baby’s ethnicity (white, Asian, black, other). All covariates were included in the model a priori based on previous knowledge, except for birth season, birth year, and the random intercept for middle layer super output areas which were included as they were influential in the model*.* In joint air pollutant-noise models we further adjusted air pollutants for noise, and vice versa. We ran two air pollutant models for term LBW and continuous term birth weight, assessing models on a case by case basis for collinearity by inspecting the variance inflation factor and standard errors. We also evaluated the relation between exposures and term birth weight (unadjusted, adjusted, and joint exposure models) using generalised additive models to evaluate non-linearity.

We ran sensitivity analyses on joint air pollutant-noise models evaluating possible effect modification by ethnicity (interaction term for exposure multiplied by ethnicity); and excluding those exposed to aircraft or railway noise >50 dB – the latter to remove the influence of high aircraft or railway noise and allow the evaluation of the influence of road traffic noise in a cleaner subgroup.

**Fig 2:**
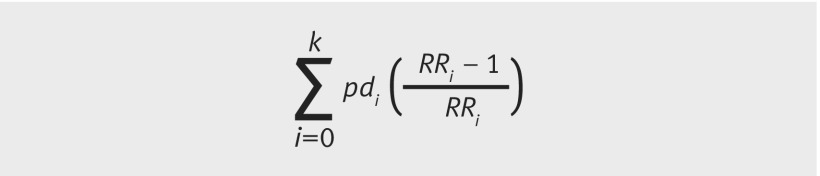
Equation. pd_i_=the proportion of cases falling into ith exposure level; RR_i_=the adjusted relative risk comparing ith exposure level with reference group (i=0)

We calculated the population attributable fraction for term LBW for exposure to PM_2.5_ greater than the 25th centile of the exposure distribution, using the formula in figure 2.^27^ The exposure levels were quartiles for this calculation. All analyses were conducted in Stata (version 13), except generalised additive models which were run in R (version 3.1.2) using the mgcv package. No adjustment for multiple testing was made.

### Patient involvement

No patients were involved in setting the research question or the outcome measures, nor were they involved in developing plans for design or implementation of the study. No patients were asked to advise on interpretation or writing up of results. There are no plans to disseminate the results of the research to study participants or the relevant patient community. 

## Results

Table 1 shows that 2.6% and 9.5% of term births were classified as LBW and SGA respectively. Over the study period of 2006 to 2010, there were temporal trends for LBW (decreasing), air pollutant exposures (decreasing particularly for PM_2.5 traffic-exhaust_, PM_2.5_, PM_10_), and an increasing proportion of births with high noise exposures, the latter reflecting change in spatial distribution of maternal addresses over time, as noise modelling was not time varying. Supplementary table 1 in web appendix 1 shows that air pollutant exposures were positively correlated (0.45 to 1.00), except with O_3_ (-0.46 to -0.77). Daytime and night-time road traffic noise were very highly correlated (∼1.00), and road traffic noise was positively correlated with air pollutant exposures (0.15 to 0.50) except O_3_ (∼-0.15). Maternal age, ethnicity, birth registration type, birth season, birth year, deprivation (Carstairs quintile), and tobacco expenditure were associated with outcomes and exposures (supplementary tables 2 and 3 in web appendix 1).

**Table 1:** Characteristics of the study population and distribution of pregnancy outcomes and exposures

Variable	No	Mean term birth weight (g)	Term LBW (%)	Term SGA* (%)		Mean pregnancy average concentration (μg/m^3^)								% Exposed ≥65 dB	
						NO_2_	NO_x_	PM_2.5 traffic exhaust_	PM_2.5 traffic non-exhaust_	PM_2.5_	PM_10_	O_3_		L_Aeq,16hr_	L_night_
Total population	540 365	3392	2.6	9.5		40.6	72.5	0.61	0.73	14.4	23.1	31.9		14.2	6.3
Infant sex:															
Male	275 546	3454	2.1	9.5		40.6	72.5	0.61	0.73	14.4	23.1	31.9		14.1	6.2
Female	264 819	3328	3.1	9.5		40.6	72.4	0.61	0.73	14.4	23.1	31.9		14.2	6.3
Maternal age (years):															
<25	100 931	3316	3.3	12.7		40.8	73.0	0.62	0.73	14.5	23.2	31.7		15.9	7.1
25-29	140 353	3369	2.8	9.7		40.5	72.3	0.61	0.73	14.4	23.1	31.9		15.3	6.9
30-34	169 559	3421	2.2	8.6		40.4	72.1	0.61	0.72	14.4	23.0	32.0		13.6	6.0
≥35	129 522	3438	2.2	8.2		40.6	72.7	0.61	0.72	14.4	23.1	31.8		12.3	5.2
Ethnicity:															
White	286 192	3470	1.7	9.6		39.8	70.5	0.59	0.70	14.3	22.9	32.3		12.9	5.5
Asian	93 555	3196	5.1	9.6		40.9	73.1	0.62	0.74	14.4	23.1	31.7		15.0	6.4
Black	91 740	3359	2.8	9.4		42.0	76.2	0.66	0.78	14.6	23.5	31.0		15.4	7.2
Other	68 878	3379	2.3			41.4	74.6	0.64	0.76	14.5	23.3	31.5		16.6	7.8
Birth registration:															
Within marriage^†^	348 157	3397	2.5	13.0		40.6	72.6	0.61	0.73	14.4	23.1	31.8		13.7	6.0
Sole registration	35 937	3329	3.4	10.3		41.3	74.4	0.64	0.75	14.6	23.3	31.4		15.7	7.4
Joint with same address	105 239	3425	2.2	12.9		40.0	70.9	0.60	0.71	14.4	23.0	32.3		14.8	6.7
Joint with different address	51 032	3339	3.2	9.5		40.8	73.1	0.62	0.74	14.4	23.1	31.7		14.6	6.5
Birth season:															
Winter	130 033	3382	2.7	9.7		39.6	70.1	0.61	0.71	13.9	22.6	31.0		14.4	6.4
Spring	133 395	3390	2.6	9.4		43.3	79.6	0.68	0.78	14.8	23.8	27.4		13.9	6.0
Summer	138 418	3399	2.5	9.3		42.0	76.2	0.63	0.75	15.0	23.8	32.8		14.1	6.3
Autumn	138 519	3398	2.5	9.5		37.4	64.0	z	0.66	13.9	22.3	36.1		14.3	6.4
Birth year:															
2006	101 770	3382	2.8	9.7		42.3	77.6	0.72	0.71	16.1	25.1	30.4		13.7	6.0
2007	106 528	3388	2.6	9.4		40.6	71.7	0.63	0.69	14.8	24.1	34.2		13.9	6.1
2008	106 678	3394	2.6	9.3		42.1	77.7	0.63	0.76	14.5	23.5	30.6		14.1	6.2
2009	110 014	3397	2.5	9.1		41.0	73.1	0.60	0.77	14.0	22.6	27.4		14.2	6.3
2010	115 375	3398	2.4	9.5		37.3	63.2	0.51	0.69	12.9	20.5	36.5		14.8	6.6
Carstairs quintile:															
1st, least deprived	85 358	3467	1.6	8.9		37.3	64.2	0.51	0.61	14.1	22.5	33.7		9.2	2.6
2nd	92 264	3433	2.0	9.4		39.3	69.3	0.57	0.69	14.3	22.8	32.6		13.6	5.6
3rd	100 934	3400	2.4	10.1		40.4	72.0	0.60	0.72	14.4	23.0	32.0		15.2	6.6
4th	119 239	3368	2.9	10.7		41.1	73.8	0.63	0.75	14.5	23.2	31.5		15.9	7.2
5th, most deprived	142 570	3335	3.3	9.5		43.0	78.6	0.70	0.81	14.7	23.6	30.5		15.3	7.9
Tobacco expenditure quintile:															
1st	110 332	3436	1.9	8.3		37.9	65.7	0.52	0.63	14.2	22.6	33.3		10.5	3.2
2nd	110 146	3415	2.3	9.0		40.1	71.3	0.59	0.71	14.4	23.0	32.1		13.5	5.5
3rd	109 477	3389	2.6	9.6		41.0	73.6	0.63	0.75	14.5	23.2	31.6		16.2	7.6
4th	109 499	3362	3.0	10.3		41.0	73.5	0.63	0.74	14.5	23.2	31.7		16.2	7.8
5th	100 911	3354	3.1	10.7		42.9	78.6	0.70	0.80	14.7	23.6	30.6		14.5	7.4
London region:															
Inner	173 181	3395	2.5	9.4		45.1	84.3	0.78	0.88	14.8	24.0	29.4		17.1	9.4
Outer	367 184	3391	2.6	9.5		38.4	66.9	0.54	0.65	14.2	22.7	33.1		12.8	4.8

LBW=low birth weight (<2500 g); SGA=small for gestational age *SGA, % out of a total 471 489 for whom sex-ethnicity specific SGA calculated †Includes civil partnerships

### Air pollution

**Fig 3:**
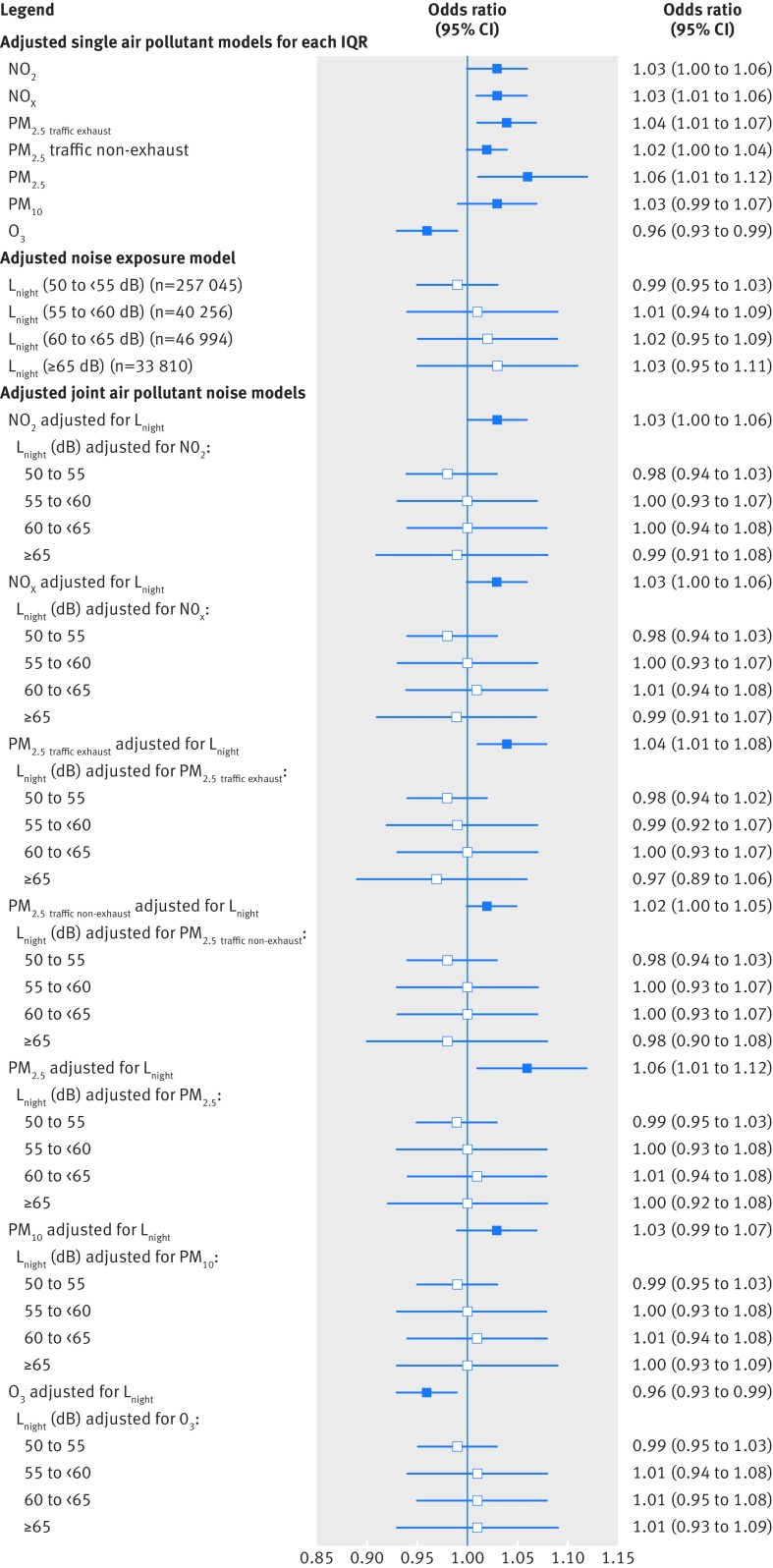
Odds of term low birth weight (LBW) associated with air pollutants (for each interquartile range (IQR)) and night-time noise (L_night_) in single exposure and joint exposure models. Odds ratios for night-time noise (L_night_) are versus the reference group <50 dB. All models are adjusted for sex, maternal age, ethnicity, birth registration type, birth season, birth year, Carstairs quintile (census output area level), tobacco expenditure (census output area level), gestational age as linear and quadratic terms, and random intercept for middle layer super output areas, in addition to including the air pollutant or noise metrics shown above. IQR values for air pollutants: NO_2_ (for each IQR, 8.6 μg/m^3^), NO_x_ (23.7 μg/m^3^), PM_2.5 traffic exhaust_ (0.35 μg/m^3^), PM_2.5 traffic non-exhaust_ (0.29 μg/m^3^), PM_2.5_ (2.2 μg/m^3^), PM_10_ (3.0 μg/m^3^), and O_3_ (8.4 μg/m^3^)

Figure 3 and supplementary tables 4 to 6 in web appendix 1 show that in single pollutant adjusted models, IQR increases in exposure to primary pollutants related to traffic (NO_2_, NO_x_, PM_2.5 traffic exhaust_, PM_2.5 traffic non-exhaust_), PM_2.5_, and PM_10_ during pregnancy were associated with 2% to 6% increased odds of term LBW (eg, odds ratios of 1.03, 95% confidence interval 1.00 to 1.06 for NO_2_; and 1.04, 1.01 to 1.07 for PM_2.5 traffic exhaust_), 1% to 3% increased odds of term SGA, and reduced term birth weight. Figure 3 shows that decreased odds of term LBW were observed with increasing O_3_ exposure. Consistent with this, in adjusted generalised additive models, term birth weight decreased approximately linearly with increasing exposure to air pollutants (except O_3_) (not shown).

**Fig 4:**
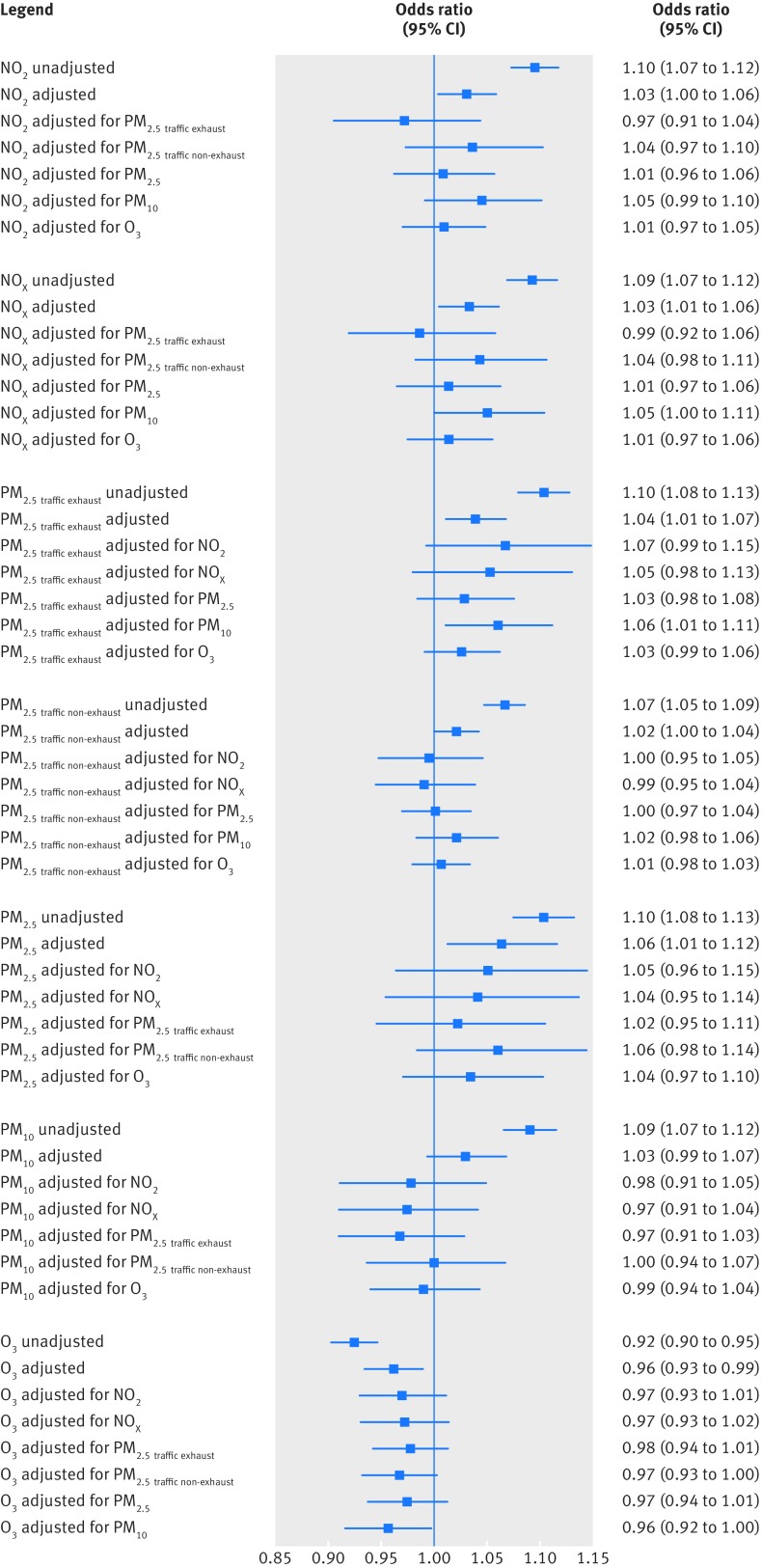
Odds of term low birth weight (LBW), associated with interquartile range (IQR) increases in air pollutants, in single and two air pollutant models. Adjusted models are adjusted for sex, maternal age, ethnicity, birth registration type, birth season, birth year, Carstairs quintile (census output area level), tobacco expenditure (census output area level), gestational age as linear and quadratic terms, and random intercept for middle layer super output areas, in addition to including the air pollutant shown above. NO_2_ and NO_x_ were not entered into the same model together as they were too highly correlated. PM_2.5_ and PM_10_ were not entered into the same model together as PM_2.5_ is a substantial subset of PM_10_ (>50% by mass). IQR values for air pollutants: NO_2_ (for each IQR, 8.6 μg/m^3^), NO_x_ (23.7 μg/m^3^), PM_2.5 traffic exhaust_ (0.35 μg/m^3^), PM_2.5 traffic non-exhaust_ (0.29 μg/m^3^), PM_2.5_ (2.2 μg/m^3^), PM_10_ (3.0 μg/m^3^), and O_3_ (8.4 μg/m^3^)

Figure 4 shows that in two air pollutant models, only PM_2.5 traffic exhaust_ and PM_2.5_ consistently had odds ratios above one associated with term LBW when adjusted, in turn, for other air pollutants. Reduced term birth weight was consistently associated with PM_2.5 traffic-exhaust_ only (supplementary figure 2 in web appendix 1). We checked two air pollutant models for multicollinearity on a case by case basis. Models with very high variance inflation factors were excluded (eg, PM_2.5 traffic exhaust_ and PM_2.5 traffic non-exhaust_), and where variance inflation factor values were borderline around 10, we excluded the model if the standard error more than doubled. However, for all two air pollutant models presented there was some increase in the standard errors for the exposure terms, which reflects the correlation structure between pollutants.

### Noise

Figure 3 and supplementary tables 4 and 5 in web appendix 1 show that in adjusted models, high (≥65 dB) night-time road traffic noise exposure was associated with an odds ratio of 1.03 (95% confidence interval, 0.95 to 1.11) for term LBW, and 1.03 (0.99 to 1.08) for term SGA, compared with the reference group (<50 dB), with a suggestion of increasing odds ratios across increasing night-time noise categories for term LBW. There was a suggestion of an exposure-response relation of decreasing term birth weight across increasing night-time and daytime road traffic noise categories (supplementary table 6 in web appendix 1). In adjusted generalised additive models, term birth weight decreased with increasing exposure to road traffic noise in a largely linear fashion (not shown).

### Air pollution and noise

**Fig 5:**
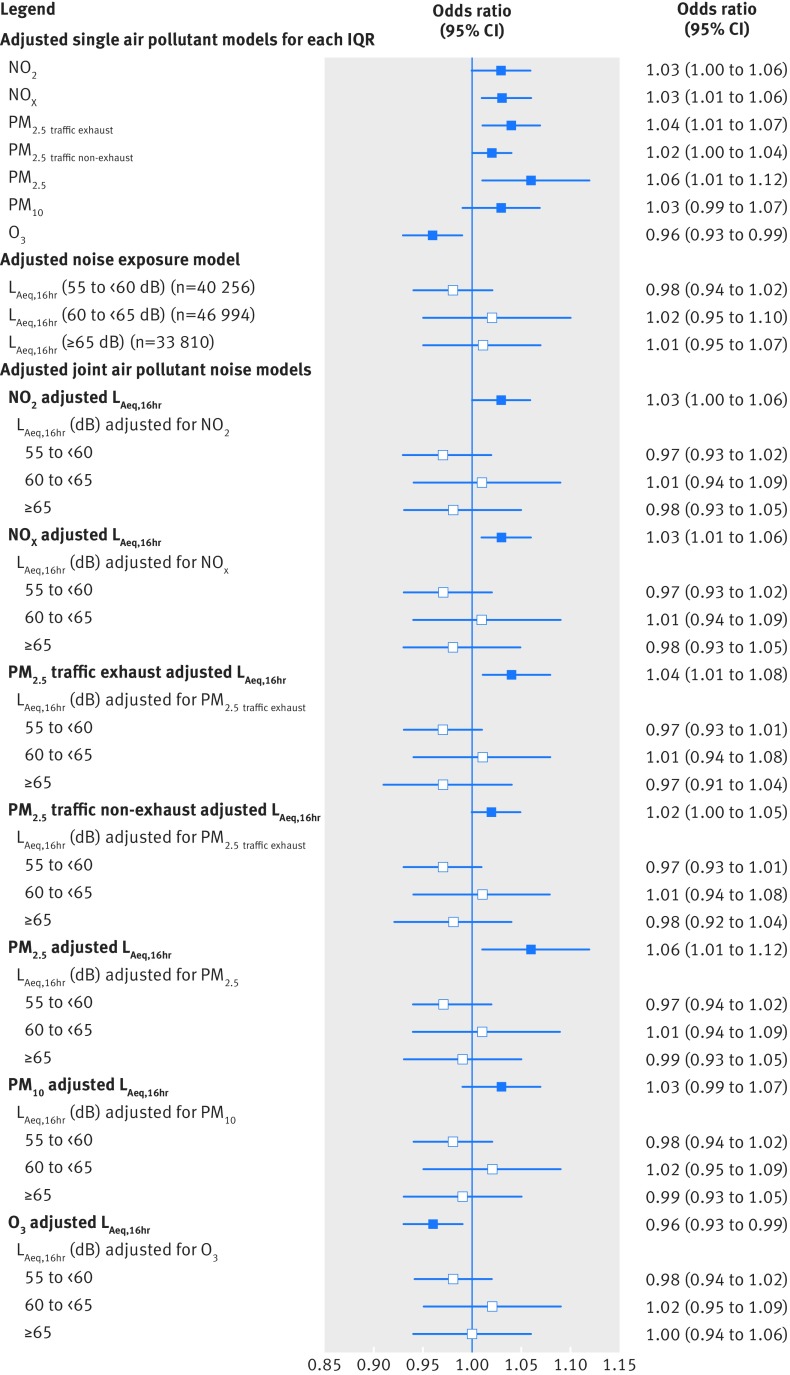
Odds of term LBW associated with air pollutants (for each interquartile range (IQR)) and daytime noise (L_Aeq,16hr_), in single exposure and joint exposure models. All noise odds ratios are versus the reference group <55 dB. All models are adjusted for sex, maternal age, ethnicity, birth registration type, birth season, birth year, Carstairs quintile (census output area level), tobacco expenditure (census output area level), gestational age as linear and quadratic terms, and random intercept for middle layer super output areas, in addition to including the air pollutant or noise metrics shown above. IQR values for air pollutants: NO_2_ (for each IQR, 8.6 μg/m^3^), NO_x_ (23.7 μg/m^3^), PM_2.5 traffic exhaust_ (0.35 μg/m^3^), PM_2.5 traffic non-exhaust_ (0.29 μg/m^3^), PM_2.5_ (2.2 μg/m^3^), PM_10_ (3.0 μg/m^3^), and O_3_ (8.4 μg/m^3^)

**Fig 6:**
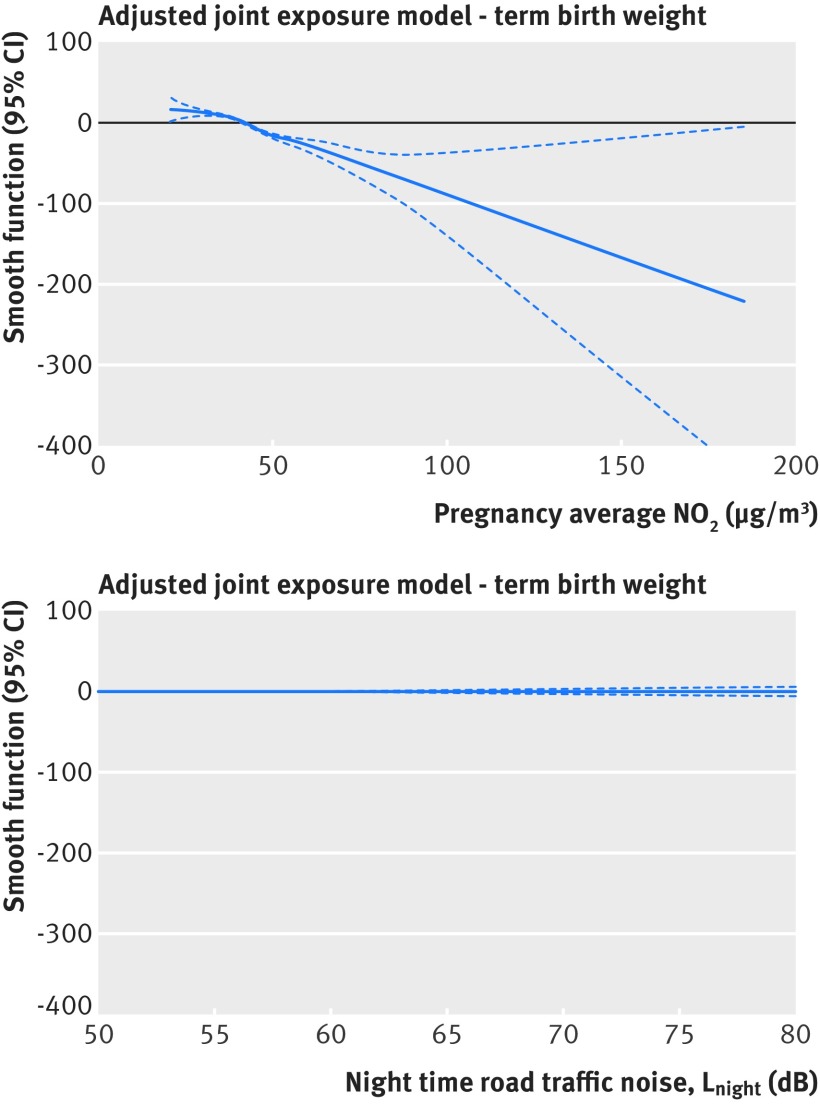
Adjusted generalised additive model for NO_2_ and L_night_. The plots show smoothing functions with 95% confidence intervals for the association between term birth weight and NO_2_ and night-time noise (L_night_) in joint exposure models. The model is adjusted for sex, maternal age, ethnicity, birth registration type, birth season, birth year, Carstairs quintile (census output area level), tobacco expenditure (census output area level), and gestational age as linear and quadratic terms

Figures 3 and 5 and table 2 show that air pollutant associations with term LBW were robust to adjustment for night-time or daytime road traffic noise, with virtually no change to odds ratios. The same holds for term SGA (table 2) and term birth weight (supplementary figures 3 and 4 and supplementary table 7 in web appendix 1). Air pollutant effect estimates adjusted for noise as a continuous variable (for each IQR) (supplementary table 8 in web appendix 1) were virtually identical to those from the primary analysis which adjusted for noise as a categorical variable. Consistent with the linear regression models, in adjusted joint exposure generalised additive models, air pollution associations with term birth weight were robust to adjustment for road traffic noise. Figure 6 shows the joint model for NO_2_ and night-time noise (L_night_), with the remaining models in web appendix 1.

**Table 2:** Joint air pollutant-noise models

Exposure	Term LBW				Term SGA		
	No	Odds ratio (95% CI)	P value*		No	Odds ratio (95% CI)	P value*
Air pollutant (for each IQR), adjusted for night-time noise:							
NO_2_	540 365	1.03 (1.00 to 1.06)			471 489	1.01 (0.99 to 1.03)	
NO_x_	540 365	1.03 (1.00 to 1.06)			471 489	1.01 (0.99 to 1.03)	
PM_2.5 traffic exhaust_	540 365	1.04 (1.01 to 1.08)			471 489	1.02 (1.00 to 1.04)	
PM_2.5 traffic non-exhaust_	540 365	1.02 (1.00 to 1.05)			471 489	1.01 (0.99 to 1.02)	
PM_2.5_	540 365	1.06 (1.01 to 1.12)			471 489	1.03 (1.00 to 1.06)	
PM_10_	540 365	1.03 (0.99 to 1.07)			471 489	1.00 (0.98 to 1.03)	
O_3_	540 365	0.96 (0.93 to 0.99)			471 489	0.99 (0.98 to 1.01)	
Night-time noise, L_night_, adjusted for NO_2_:							
<50 dB	162 260	Reference			142 880	Reference	
50 to <55 dB	257 045	0.98 (0.94 to 1.03)			224 864	1.00 (0.97 to 1.02)	
55 to <60 dB	40 256	1.00 (0.93 to 1.07)			34 960	1.02 (0.98 to 1.06)	
60 to <65 dB	46 994	1.00 (0.94 to 1.08)			40 344	1.00 (0.96 to 1.04)	
≥65 dB	33 810	0.99 (0.91 to 1.08)			28 441	1.02 (0.97 to 1.07)	
P value for trend			0.962				0.432
Air pollutant (for each IQR), adjusted for daytime noise:							
NO_2_	540 365	1.03 (1.00 to 1.06)			471 489	1.01 (1.00 to 1.03)	
NO_x_	540 365	1.03 (1.01 to 1.06)			471 489	1.01 (1.00 to 1.03)	
PM_2.5 traffic exhaust_	540 365	1.04 (1.01 to 1.08)			471 489	1.02 (1.01 to 1.04)	
PM_2.5 traffic non-exhaust_	540 365	1.02 (1.00 to 1.05)			471 489	1.01 (0.99 to 1.02)	
PM_2.5_	540 365	1.06 (1.01 to 1.12)			471 489	1.03 (1.00 to 1.06)	
PM_10_	540 365	1.03 (0.99 to 1.07)			471 489	1.01 (0.98 to 1.03)	
O_3_	540 365	0.96 (0.93 to 0.99)			471 489	0.99 (0.98 to 1.01)	
Daytime noise, L_Aeq,16hr_, adjusted for NO_2_:							
<55 dB	157 491	Reference			138 696	Reference	
55 to <60 dB	265 603	0.97 (0.93 to 1.02)			232 346	0.99 (0.96 to 1.01)	
60 to <65 dB	40 755	1.01 (0.94 to 1.09)			35 334	1.01 (0.97 to 1.05)	
≥65 dB	76 516	0.98 (0.93 to 1.05)			65 113	0.99 (0.96 to 1.03)	
P value for trend			0.802				0.957

LBW=low birth weight; SGA=small for gestational age; IQR=interquartile range *P value for linear trend across increasing noise categories.

Models are adjusted for sex (term LBW model only), maternal age, ethnicity (term LBW model only), birth registration type, birth season, birth year, Carstairs quintile (census output area level), tobacco expenditure (census output area level), gestational age as linear and quadratic terms, and random intercept for middle layer super output areas, in addition to including the air pollutant or noise metrics shown above. All air pollution estimates are adjusted for either night-time (L_night_) or daytime noise (L_Aeq,16hr_) as specified in the table, and noise estimates are adjusted for traffic related air pollution exposure (NO_2_). IQR values for air pollutants: NO_2_ (for each IQR, 8.6 μg/m^3^), NO_x_ (23.7 μg/m^3^), PM_2.5 traffic_ _exhaust_ (0.35 μg/m^3^), PM_2.5 traffic non-exhaust_ (0.29 μg/m^3^), PM_2.5_ (2.2 μg/m^3^), PM_10_ (3.0 μg/m^3^), and O_3_ (8.4 μg/m^3^)

After adjustment for each air pollutant, in turn, there was no evidence that increasing night-time or daytime road traffic noise exposure (analysed as either a categorical or continuous variable) was associated with increasing risk of term LBW (figs 3 and 5) or term SGA (supplementary tables 8 and 9 in web appendix 1). There was some suggestion of an association with reduced term birth weight in the highest night-time road traffic noise category after adjustment for NO_2_ or NO_x_ but not after adjustment for PM_2.5 traffic exhaust_ or PM_2.5 traffic non-exhaust_. However, this was not evident in adjusted joint exposure generalised additive models – which indicated that once adjusted for any of the primary traffic related air pollutants, in turn, there appears to be no relation between road traffic noise and term birth weight (fig 6 and web appendix 1). A weak association remained between road traffic noise and reduced term birth weight after adjustment for PM_2.5_, PM_10_, and O_3_ in linear regression (supplementary figure 3 and supplementary tables 8 and 9 in web appendix 1) and generalised additive models (web appendix 1).

### Trimester specific air pollution models

For term LBW, odds ratios for primary traffic related air pollutant exposures in the second and third trimesters tended to be stronger than for first trimester exposures (supplementary table 10 in web appendix 1). Conversely, for term SGA, odds ratios for exposures in earlier trimesters were stronger than the third trimester exposure for PM_2.5 traffic exhaust_ and PM_2.5 traffic non-exhaust_, and first trimester exposure appeared to be strongest for PM_2.5_ and PM_10_ (supplementary table 10 in web appendix 1). However, confidence intervals for trimester specific effects overlapped. These analyses are presented according to prespecified pollutant specific increments (not IQR) to allow comparison between trimesters for each pollutant.

### Additional analyses

Compared with unadjusted analyses (supplementary tables 4 to 6 in web appendix 1), effect sizes were generally reduced in single or joint pollutant adjusted models. Given the strong relation between exposures and census output area level deprivation, we ran birth weight models without adjustment for Carstairs quintile to check for overadjustment, however, there were only small changes in birth weight coefficients (<1 g) and the pattern of results was unchanged (not shown). The inclusion of a random intercept for middle layer super output areas (to models adjusted for all other covariates described) resulted in relatively small changes to associations for noise, term LBW, or SGA, but considerable attenuation of associations between air pollutants and term birth weight (-18% to -28% for primary traffic related air pollutants, and -35% to -49% for pollutants including regional or urban background contributions).

All sensitivity analyses were conducted on joint air pollutant-noise models. Noise analyses were largely unchanged after excluding those exposed to aircraft or rail noise greater than 50 dB (not shown). We did not observe interactions between ethnicity and air pollution or road traffic noise exposures for term LBW or SGA. Ethnicity-exposure interactions were observed in term birth weight analyses with both primary traffic related air pollutants (P value<0.001) and road traffic noise exposures (∼0.028 for daytime noise, 0.005 for night-time noise), with inverse relations for primary traffic related air pollutants across all ethnic strata (supplementary table 11 in web appendix 1).

The population attributable fraction estimated for term LBW for exposure to PM_2.5_ over the 25th centile of the distribution (ie, 13.8 μg/m^3^) during pregnancy was 3% (0% to 7%). This 3% corresponds to 93 (0-216) cases of term LBW out of a total of 2950 cases each year on average in our London study population which are directly attributable to residential exposure during pregnancy to PM_2.5_>13.8 μg/m^3^.

## Discussion

To our knowledge, this is the largest UK study on air pollution and birth weight, and the first UK study and largest study worldwide of birth weight and noise exposure. We observed that long term exposure during pregnancy to NO_2_, NO_x_, PM_2.5_ overall and specifically from traffic exhaust and non-exhaust sources, and PM_10_, were all associated with increased risk of LBW at term, across London. There was strong confounding of the relation between road traffic noise and birth weight by primary traffic related air pollutant coexposures, and our results, particularly from generalised additive models, suggest little evidence for an independent exposure-response effect of traffic related noise on birth weight outcomes. Our findings from two air pollutant models suggest that associations between term LBW and air pollutants emitted from vehicle exhausts may be driven by the fine particulate matter (PM_2.5 traffic exhaust_) component rather than the gaseous NO_x_ component.

### Strengths and weaknesses of this study

This study benefits from highly spatially resolved air pollution modelling assigned at address level, and noise levels estimated at address point. For noise particularly, this represents an advance on previous studies which have assigned noise exposure with lower spatial precision (eg, at postcode level,^8^ or according to 50 m or 250 m buffers around maternal address,^7^ or based on road proximity)^28^, and consequently reduces potential exposure misclassification, as noise levels may change dramatically over short distances (tens of metres). Nonetheless, the potential for exposure misclassification remains. For air pollution, there may be some exposure misclassification close to sources (where gradients of primary pollutants are steep). However, most people do not live within 10 m to 30 m of the centre of a main road so the impact on this study will be low. The percentage of maternal residences in our dataset within 10 m of a major road (annual average daily traffic (AADT) >10 000 vehicles) was 0.07%, within 20 m was 5%, and within 30 m was 11%. We examined the relation between living within 10 m, 20 m, and 30m of a major road and key individual level variables (ethnicity, birth registration type, and maternal age). These variables were not associated with living within 10 m of a major road. The percentage of mothers living within either 20 m or 30 m of a major road was slightly greater (by up to 3%) for non-white ethnicities (*v* white), unmarried mothers (*v* married), and younger (*v* older) maternal age groups. However, these percentage differences are very small (≤3%), so there is no reason to assume that this would introduce serious bias. Most importantly, however, whilst there may be some exposure misclassification between the exposure at the actual address versus the grid point estimate assigned, this should introduce no bias because we have assigned the nearest 20 m × 20 m point. To introduce bias we would always have to choose the point on the side of the residence closest to the road and this is unlikely. 

The air pollutant model predicted PM_2.5_ and PM_10_ slightly more accurately than NO_2_ and NO_x_, but the model bias was in the same direction (over prediction) for all these pollutants. Greater model prediction uncertainty for NO_2_ and NO_x_ may result in effect estimates for NO_2_ and NO_x_ being more conservative than those for PM_2.5_ and PM_10_ and therefore may limit our ability to directly compare the magnitude of effect estimates for NO_2_ or NO_x_ with PM_2.5_ or PM_10_. 

The noise model is likely to have overestimated and underestimated noise on some minor roads (owing to the constant for traffic on minor roads), but there is no geographical pattern (ie, autocorrelation) in any bias as a result of this,^17^ however, to reduce potential exposure misclassification we categorised noise exposure for analysis. We avoided selection bias by using all birth registration data. Direct measures of individual level smoking or deprivation data were unavailable, but we adjusted for tobacco expenditure and deprivation (Carstairs quintile) at census output area level, as in previous epidemiological studies.^29^ ^30^ We have also adjusted for birth registration type, an individual level variable which relates to both individual level qualifications and housing tenure (and thus socioeconomic status or deprivation) and individual level smoking.^31^ We cannot exclude the possibility of some residual confounding by maternal smoking, passive smoking, or deprivation, but we have adjusted for deprivation and smoking by proxy at individual level, in addition to at area level. Information on parity was not available as part of this study, so we could not adjust for any potential confounding effects directly, but an association between parity and exposure is most likely through deprivation (at area level or individual level), ethnicity, or maternal age, and these have been adjusted for. There is some evidence to suggest that extremes of ambient temperature may be associated with adverse birth outcomes (eg, preterm birth or early delivery and LBW).^32^ ^33^ ^34^ ^35^ ^36^ Meteorological conditions, including ambient temperature, are related to air pollution levels. By adjusting for season we did adjust for general seasonal variation in average temperatures, but we could not adjust for exposure to extreme ambient temperatures as we did not have data on temperature linked to the births data. We could not account for residential mobility during pregnancy (∼16% in UK^37^), nor exposures away from maternal residence (eg, workplace or transport), indoor air pollution, or exposure modification owing to behaviours (eg, opening windows), or building characteristics (eg, bedroom façade). These could contribute to exposure misclassification. 

We were not able to adjust for spontaneous versus medical intervention early delivery (which could influence the outcome indirectly by gestation period), as data on delivery type were not available as part of this study from the birth registry or NHS Numbers for Babies (NN4B) datasets. If clinical practice in medical intervention for early delivery varies spatially (eg, between hospitals or owing to cultural factors), this could potentially confound the spatial component of exposure metrics. However, all our epidemiological models included a random effect for small area (middle layer super output areas – average population 8000) specifically to account for underlying spatial patterns in the data, so we do not think this should be a serious issue. Multiple hypothesis tests were performed, so the multiple testing problem (ie, that the probability of a Type 1 error will be greater than 0.05 (5%)), should be considered when interpreting P values.

### Strengths and weaknesses in relation to other studies

Our single air pollutant model findings are consistent with recent meta-analyses which report increased risk of low birth weight (LBW) and reduced mean birth weight associated with NO_2_,^1^ PM_2.5_,^38^ and PM_10_.^1^ ^39^ Meta-analysis results for O_3_ are less clear: odds ratio for LBW of 1.01 (95% confidence interval, 0.82 to 1.25) for each 20 ppb increase in pregnancy exposure to O_3_.^1^ To our knowledge, only three Californian studies, have examined source specific PM_2.5_ and birth weight. Converted to the same interquartile range (IQR) (0.35 μg/m^3^) scale as our PM_2.5 traffic exhaust_ analyses, these studies each report 2% increased odds of term LBW for PM_2.5_ from diesel and 3% to 4% increased odds for PM_2.5_ from gasoline,^40^ ^41^ ^42^ consistent in magnitude with our odds ratio for term LBW of 1.04 (95% confidence interval, 1.01 to 1.07) for each IQR increase. To our knowledge, no previous study has reported two pollutant models including source specific PM_2.5_. Our findings, that only PM_2.5 traffic exhaust_ (out of PM_2.5 traffic exhaust_, NO_2_, and NO_x_) showed a consistent elevated risk with mutual adjustment, suggesting that associations between LBW and air pollutants emitted from vehicle exhausts may be driven by the fine particulate matter (PM_2.5 traffic exhaust_) component rather than the gaseous NO_x_ component is an important and new contribution to scientific knowledge. Our study also shows associations between LBW and fine particulate matter from road traffic which is not emitted from the vehicle exhaust (ie, brake or tyre wear particles and vehicle induced resuspension of road dust). However, owing to multicollinearity in models containing both PM_2.5 traffic exhaust_ and PM_2.5 traffic non-exhaust_, we could not separate potential effects of traffic related exhaust and non-exhaust related PM_2.5_. The magnitude of association with PM_2.5 traffic exhaust_ was consistently stronger than with PM_2.5 traffic non-exhaust_, and this could reflect differing chemical constituents (and thus toxicity) of the PM_2.5_ mixture from different sources.

We found that associations between road traffic noise and term birth weight were strongly attenuated when adjusted for primary air pollutants related to traffic: to null when adjusted for PM_2.5 traffic exhaust_ or PM_2.5 traffic non-exhaust_, although after adjustment for NO_2_ or NO_x_ an association between night-time noise and reduced birth weight in the highest exposure category remained, which could possibly reflect a threshold effect. The results of our generalised additive models adjusted for NO_2_ or NO_x_, however, do not support an independent association with road traffic noise, or suggest any threshold effect for noise. The most recent systematic review of noise exposure and birth weight found “evidence supportive of associations between LBW and noise exposure” particularly for very high noise levels, but the evidence was inconsistent,^4^ based on 10 occupational studies, four aircraft noise studies, and two traffic noise studies. Three previous studies have examined long term air pollution and noise exposures jointly.^7^ ^8^ ^9^ Our findings are consistent with a small cohort study (n=6438) in Barcelona, which suggested elevated risks of term LBW and small for gestational age (SGA) associated with noise and air pollution exposures in single exposure adjusted models, but in a joint exposure model term LBW risk was associated with third trimester PM_2.5_ (for each 3.6 μg/m^3^, odds ratio 1.31, 95% confidence interval 1.07 to 1.61), but not noise (for each 6.7 dB (A-weighted), 0.89, 0.71 to 1.12).^7^ Term birth weight was not associated with NO_2_, NO_x_, or road traffic noise, in either fully adjusted single exposure models or joint exposure models in the Danish National Birth Cohort (n=75 166).^9^

Our findings contrast with a registry based study in Vancouver (n=68 238), which found associations between all transportation (road traffic, railway, and aircraft) noise (L_den_) and reduced term birth weight or LBW which remained after adjustment for PM_2.5_, PM_10_, and primary road traffic air pollution (NO_2_ and NO_x_), however, associations for air pollutants were attenuated to null by adjustment for transportation noise.^8^ Road traffic noise showed similar associations with term birth weight or LBW in single exposure models, but road traffic noise adjusted for air pollution was not analysed.^8^ We, however, found an association between the road traffic noise and reduced birth weight remained after adjustment for PM_2.5_ or PM_10_ (which include regional and urban background contributions) – one possible explanation is that adjusting for PM_2.5_ or PM_10_ did not fully control for confounding of noise by air pollution coexposures from road traffic. This should be noted by other researchers investigating potential health effects of road traffic noise. 

Compared with London, the noise distribution in Vancouver was wider (L_den_ mean 60.2 dB(A-weighted), range 6.2-89.0), mean air pollution exposures were lower and with less contrast in Vancouver (PM_2.5_ mean 4.1 μg/m^3^, range 0-11.3; NO_2_ mean 33.7 μg/m^3^, range 0-64.5) and Denmark (NO_2_ median 11.0 μg/m^3^, 5th-95th centiles 7.1–26.3), and air pollutant-noise correlations were lower in Vancouver (correlations with road traffic noise: 0.05 for NO_2_, 0.09 for PM_2.5_; and all transportation noise: 0.18 for NO_2_, 0.16 for PM_2.5_), but higher in Denmark (0.47 between NO_2_ and road traffic noise).^8^ ^9^ These differences, which could reflect differences in pollutant sources, may contribute to the contrasting findings from Denmark and Vancouver compared with our study. In our study the noise model floor means that the minimum modelled value of night-time noise from road traffic in London was 42.4 dB,^17^ which is higher than the recommended upper limit of exposure of total noise of 40 dB proposed by the Night Noise Guidelines for Europe.^43^ It is possible that we did not have a sufficiently low noise exposure reference group, to detect small associations between noise and birth weight, above the guideline level.

In the broader context, our findings contrast with reviews of joint air pollution and noise studies which suggest independent effects of road traffic noise on other health outcomes (eg, cardiovascular outcomes), after adjustment for air pollution.^44^ ^45^ This could reflect different biological pathways between noise and fetal growth versus other health outcomes at later stages of life. The fetus has no direct exposure to the environment, but exposure is mediated through the mother and placenta, and this may modify effects. Threshold effects may be relevant for exposure to noise, and the threshold could vary between health outcomes, possibly being higher for effects on birth weight versus, for example, cardiovascular outcomes. Alternatively, it might reflect differences between studies in the ability to control for confounding by air pollution from road traffic specifically. We did note that associations between noise and birth weight were more strongly attenuated by adjustment for primary road traffic-related air pollutants (NO_2_, NO_x_, PM_2.5 traffic exhaust_, PM_2.5 traffic non-exhaust_) compared with background air pollutants (PM_2.5_ and PM_10_). This suggests that adjusting for the background pollutants may not fully adjust for the confounding effects of air pollution coexposures directly from road traffic, in our study. With respect to cardiovascular outcomes, it has been noted that “more studies using air pollution indicators specific to road traffic are needed to properly assess if road noise and pollutant effects on CV outcomes are subjected to the confounding effect of one another.”^45^

Our results did not give a clear indication as to which trimester could be most influential with respect to air pollution and fetal growth, and previous study findings have been inconsistent on this point. The most recent meta-analyses are suggestive overall of stronger associations for later trimesters between LBW or reduced birth weight and PM_2.5_ and PM_10_,^38^ ^39^ but unclear for NO_2_.^1^ One potential explanation for this is that earlier trimester exposures may be more prone to exposure measurement bias from residential mobility (in studies assigning exposure according to maternal residential address at birth), and thus attenuated towards the null. However, there are persuasive findings from a natural experiment of air pollution reductions during the 2008 Bejing Olympics, supporting the importance of the third trimester exposures to air pollution in relation to term birth weight.^46^ This is biologically plausible, as during the third trimester the rate of fetal growth and weight gain increases dramatically and reaches its peak at about week 33.^47^ ^48^

We found effect modification by ethnicity of the relation between air pollution and reduced birth weight in line with previous studies, although results for different ethnic groups have been inconsistent.^49^ ^50^ ^51^ ^52^ ^53^ ^54^ Effect modification by ethnicity could reflect increased susceptibility to the adverse impacts of air pollution, owing to environmental inequality or differing biological susceptibility.

Biological mechanisms in which air pollution or noise may impair fetal growth are not established. Hypothesised mechanisms for air pollution are oxidative stress; endocrine disruption; changes to maternal-placental blood flow and oxygen or nutrition transfer;^55^ placental mitochondrial damage;^56^ and placental growth or function,^57^ whilst those for noise are stress triggered endocrine or immune response disruption, plasma catecholamine increase or placental blood flow decrease,^4^ hypertension,^5^ and sleep disturbance.^6^ Convincing evidence that maternal passive smoking during pregnancy is causally related to reduced birth weight,^58^ strongly supports the biological plausibility of an association between ambient air pollution and reduced birth weight, by analogy.

### Conclusion

This study suggests that in Greater London, which has 19% of all annual births in England and Wales,^59^ air pollution from road traffic is having a detrimental impact upon babies’ health, before they are born. We estimate that 3% of term LBW cases in London are directly attributable to residential exposure during pregnancy to PM_2.5_>13.8 μg/m^3^. Our results suggest little evidence for an independent exposure-response effect of traffic related noise on birth weight, but we cannot rule out that an association might be observed in a study area with a wider range of noise exposures. Our findings should be broadly generalisable to other UK and European cities or urban areas with comparable exposure levels and profiles. At city scale, environmental health policies aimed at reducing road traffic air pollution could reduce the burden of LBW, SGA, and subsequent lifelong morbidity. With the annual number of births projected to continue increasing in London,^60^ the absolute health burden will increase at the population level, unless air quality in London improves.

**What is already known on this topic**

Road traffic pollution comprises not only air pollutants such as NO_2_ and particulate matter, but also noise
There is a large body of research demonstrating associations between maternal exposure to ambient air pollution during pregnancy and reduced birth weight, low birth weight (LBW), or small for gestational age (SGA)
The relation between road traffic noise and birth weight is unclear, and research examining traffic related air pollutant and noise coexposures together is very limited, so the extent to which observed air pollution associations might be attributable to road traffic noise is poorly understood

**What this study adds**

There is an increased risk of LBW specifically in relation to the air pollution profile of London
Exposure to local air pollution from road traffic is associated with increased risk of LBW in London, but there is little evidence for an independent exposure-response effect of traffic related noise on birth weight
Reducing exposure to traffic related air pollution could reduce the burden of LBW, SGA, and subsequent morbidity, and ultimately give babies in urban environments a healthier start in life

## Web Extra

Extra material supplied by the author

## Notes

Contributors: MBT, JG, HRA, SDB, and FJK contributed to study conception and design. DF, JG, SDB, DD, HRA, and FJK contributed to exposure assessment. REG, DF, and ALH acquired health and confounder data. RBS contributed to study design, wrote the statistical analysis plan, conducted the data analyses, and drafted the initial report. MBT contributed to the statistical analysis plan, the data analyses, and initial drafting of the report. MB contributed to the study design and statistical analysis plan. All authors contributed to interpreting the analyses and critically revising the article, approved the final draft, and agree to be accountable for all aspects of the work. All authors had full access to all of the data in the study and can take responsibility for the integrity of the data and the accuracy of the data analysis. MBT is the guarantor.

Ethical approval: The study used SAHSU data (UK National Births and Stillbirth register data and NHS Numbers for Babies (NN4B)), supplied by the Office for National Statistics. The study was covered by national research ethics approval from the London-South East Research Ethics Committee (reference 17/LO/0846). Data access was covered by the Health Research Authority Confidentiality Advisory Group under Regulation 5 of the Health Service (Control of Patient Information) Regulations 2002 (reference 14/CAG/1039).

Data sharing: No additional data are available.

Transparency: The lead author, MBT, affirms that the manuscript is an honest, accurate, and transparent account of the study being reported; that no important aspects of the study have been omitted; and that any discrepancies from the study as planned have been explained.
