# Supplementary material for: Impact of London's road traffic air and noise pollution on birth weight: retrospective population based cohort study
Source: BMJ. 2017 Dec 5;359:j5299. doi: 10.1136/bmj.j5299 (PMC5712860; doi:10.1136/bmj.j5299)

Smith RB, Fecht D, Gulliver J, Beevers SD, Dajnak D, Blangiardo M, Ghosh RE, Hansell AL, Kelly FJ, Anderson HR, Toledano MB. **Impact of London's road traffic air and noise pollution on birth weight: a retrospective population-based cohort study.**

Web appendix 1: Supplementary Material

Contents

Supplementary Figure 1: The functional relationship between term birth weight and night-time (L_{night}) and day-time ($L_{Aeq,16hr}$) noise 3

Supplementary Figure 2: Mean difference in term birth weight (g), associated with IQR* increases in air pollutants, in single- and two- air pollutant models 4

Supplementary Figure 3: Adjusted mean difference in term birth weight (g), associated with air pollutants (per IQR) and night-time noise (L_{night}), in single-exposure and joint-exposure models 6

Supplementary Figure 4: Adjusted mean difference in term birth weight (g), associated with air pollutants (per IQR) and day-time noise ($L_{Aeq,16hr}$), in single-exposure and joint-exposure models 8

Supplementary Table 1: Air pollution and road traffic noise exposures in the study population (N=540,365) and their correlations 10

Supplementary Table 2: OR (95%CI) for term LBW and term SGA and mean difference (95% CI) in term birth weight (g) associated with potential confounding factors (univariate linear/logistic regression) in the study population (n=540,365) 11

Supplementary Table 3: Mean difference (95% CI) in exposure metric associated with potential confounding factors (univariate linear regression) in the study population (n=540,365) 12

Supplementary Table 4: Odds of term LBW (<2500g and ≥37 weeks gestation) associated with air pollutants and noise (OR, 95% confidence intervals) 14

Supplementary Table 5: Odds of term small-for-gestational-age (SGA) associated with air pollutants and noise (odds ratios with 95% confidence intervals) 15

Supplementary Table 6: Mean difference in term birth weight (g) associated with air pollutants and noise (with 95% confidence intervals) 16

Supplementary Table 7: Joint air-pollutant-noise models: Mean difference (95% CI) in birth weight (g) associated with air pollutants and noise amongst term births 17

Supplementary Table 8: Associations between noise on the continuous scale (per IQR) and odds of LBW and SGA and mean difference in birth weight (g) amongst term births, in unadjusted, adjusted and joint air pollutant-noise models 18

Supplementary Table 9: Joint air-pollutant-noise models: Adjusted OR (95%CI) for LBW and SGA, and mean difference (95% CI) in birth weight (g) associated with noise amongst term births, after adjustment for each air pollutant 21

Supplementary Table 10: Adjusted* odds of term LBW and term SGA associated with trimester-specific air pollutant exposures (models adjusted for road traffic noise co-exposure) 25

Supplementary Table 11: Joint air-pollutant-noise models: Adjusted mean difference (95% CI) in birth weight (g) associated with air pollutants (per IQR) and noise amongst term births 26

Supplementary Appendix 1 - Adjusted joint-exposure generalised additive models (GAMs) 27

Supplementary Figure 1: The functional relationship between term birth weight and night-time (L_{night}) and day-time ($L_{Aeq,16hr}$) noise

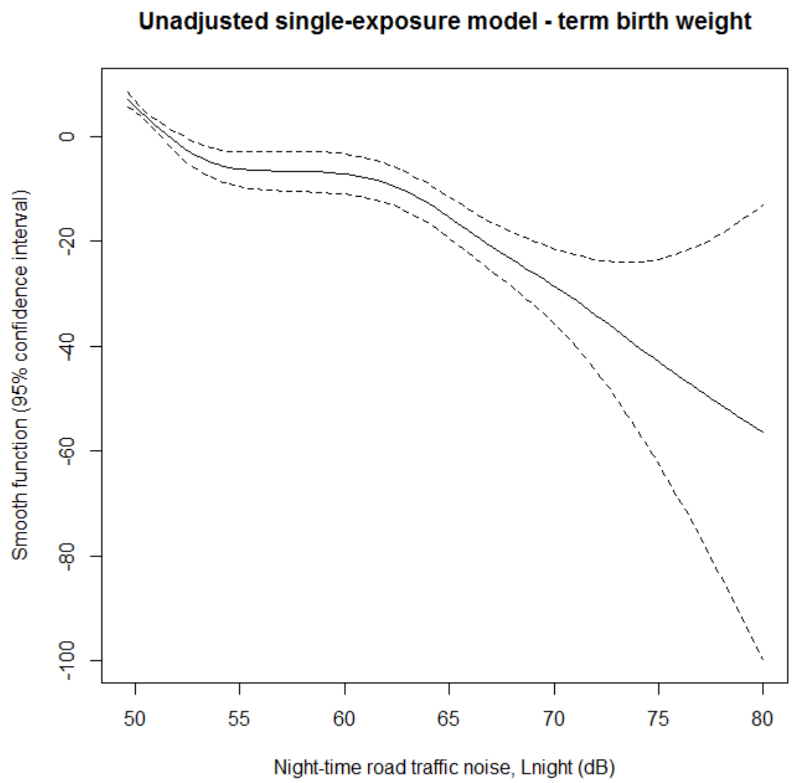

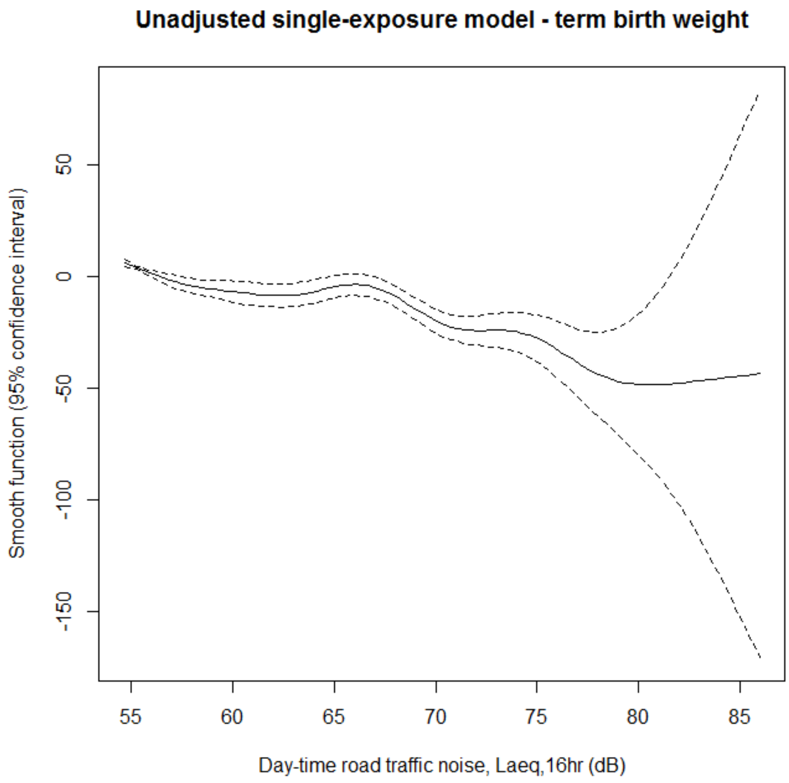

Supplementary Figure 2: Mean difference in term birth weight (g), associated with IQR* increases in air pollutants, in single- and two- air pollutant models

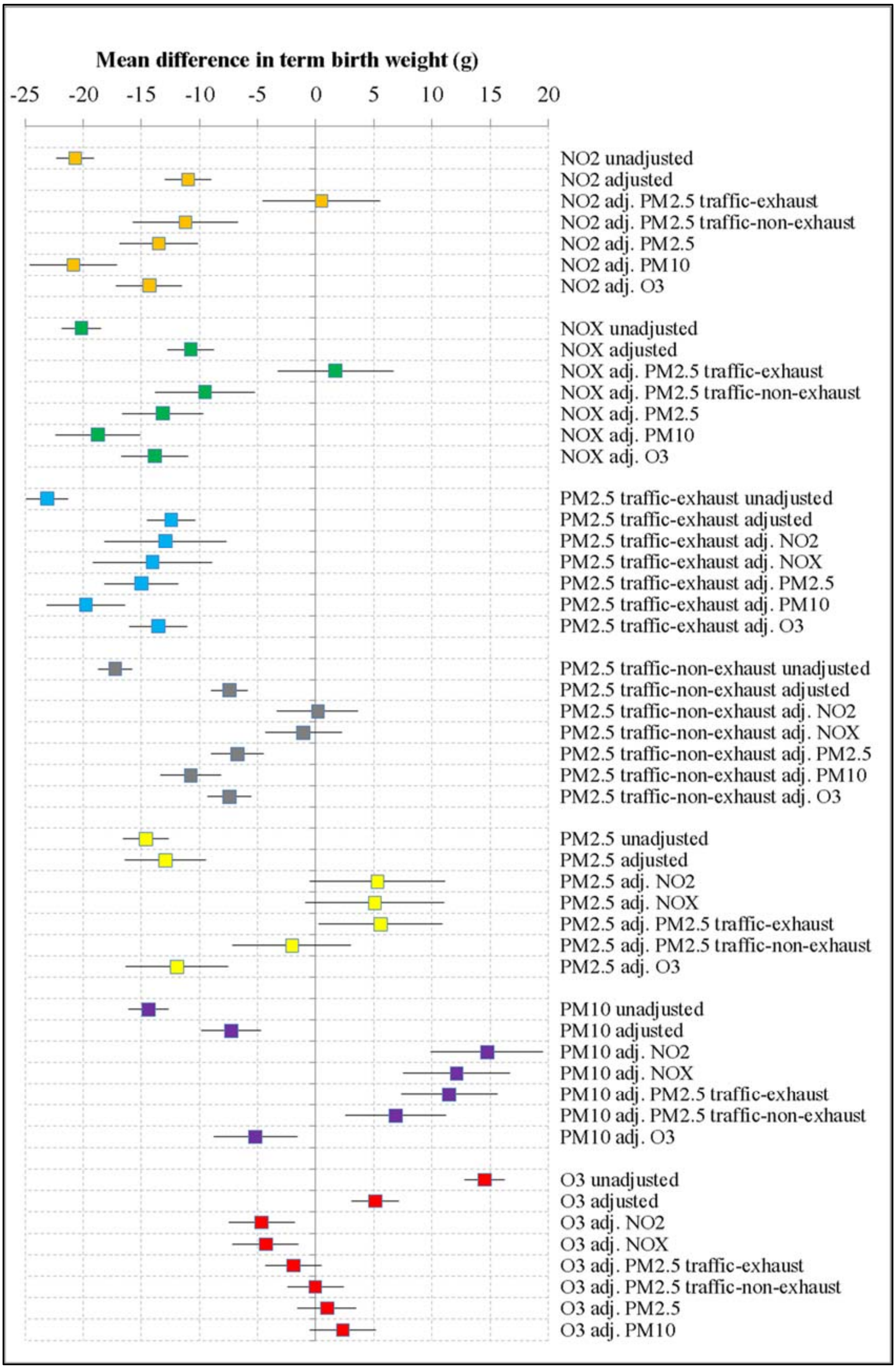

Supplementary Figure 2 footnotes: In Supplementary Figure 1 adjusted models are adjusted for sex, maternal age, birth registration type, tobacco expenditure (COA-level), Carstairs quintile (COA-level), individual-level ethnicity, gestational age as linear and quadratic terms, season of birth, year, and random intercept for MSOA in addition to including the air pollutants shown above. NO_2 and NO_X were not entered into the same model together as they were too highly correlated. $PM_{2.5}$ and PM_{10} were not entered into the same model together as $PM_{2.5}$ is a substantial subset of PM_{10} (>50% by mass). * IQR values for air pollutants: NO_2 (per IQR, 8.6 $\mu g/m^3$), NO_X (per IQR, 23.7 $\mu g/m^3$), $PM_{2.5\ traffic\ exhaust}$ (per IQR, 0.35 $\mu g/m^3$), $PM_{2.5\ traffic\ non\text{-}exhaust}$ (per IQR, 0.29 $\mu g/m^3$), $PM_{2.5}$ (per IQR, 2.2 $\mu g/m^3$), PM_{10} (per IQR, 3.0 $\mu g/m^3$), O_3 (per IQR, 8.4 $\mu g/m^3$)

Supplementary Figure 3: Adjusted mean difference in term birth weight (g), associated with air pollutants (per IQR) and night-time noise (L_{night}), in single-exposure and joint-exposure models

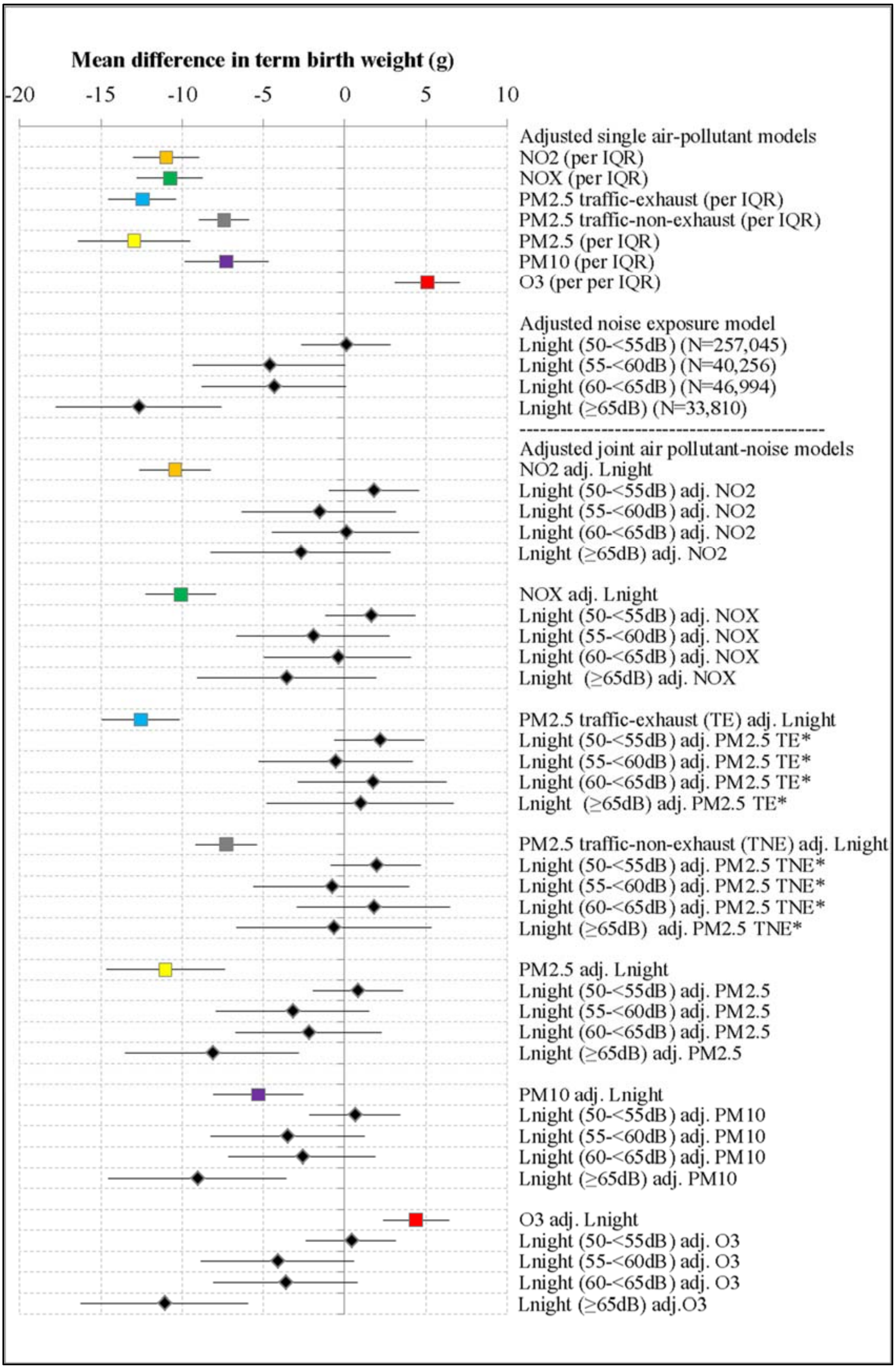

Supplementary Figure 3 footnotes: * PM2.5 TE is $PM_{2.5\ traffic\text{-}exhaust}$, and PM2.5 TNE is $PM_{2.5\ traffic\text{-}non\text{-}exhaust}$. L_{night} is night-time noise, and all noise ORs are vs. the reference group <50dB. All models in Figure 2 are adjusted for sex, maternal age, birth registration type, tobacco expenditure (COA-level), Carstairs quintile (COA-level), individual-level ethnicity, gestational age as linear and quadratic terms, season of birth, year, and random intercept for MSOA, in addition to including the air pollutant or noise metrics shown above. IQR values for air pollutants: NO_2 (per IQR, 8.6 μg/m^3), NO_X (per IQR, 23.7 μg/m^3), $PM_{2.5\ traffic\ exhaust}$ (per IQR, 0.35 μg/m^3), $PM_{2.5\ traffic\ non\text{-}exhaust}$ (per IQR, 0.29 μg/m^3), $PM_{2.5}$ (per IQR, 2.2 μg/m^3), PM_{10} (per IQR, 3.0 μg/m^3), O_3 (per IQR, 8.4 μg/m^3)

Supplementary Figure 4: Adjusted mean difference in term birth weight (g), associated with air pollutants (per IQR) and day-time noise ($L_{Aeq,16hr}$), in single-exposure and joint-exposure models

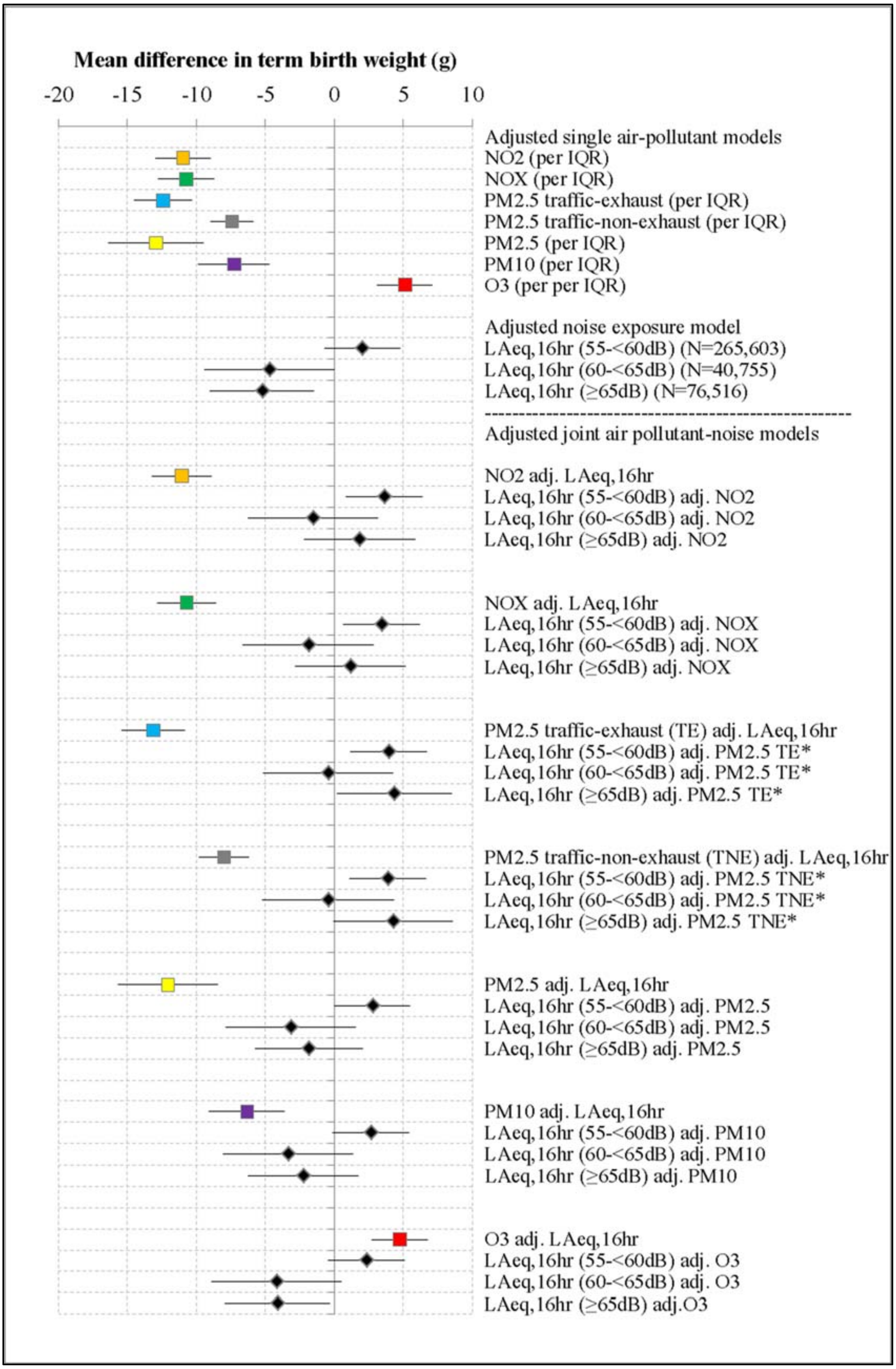

Supplementary Figure 4 footnotes: * PM2.5 TE is $PM_{2.5\ traffic\text{-}exhaust}$, and PM2.5 TNE is $PM_{2.5\ traffic\text{-}non\text{-}exhaust}$. $L_{Aeq,16hr}$ is day-time noise, and all noise ORs are vs. the reference group <55dB. All models in Figure 3 are adjusted for sex, maternal age, birth registration type, tobacco expenditure (COA-level), Carstairs quintile (COA-level), individual-level ethnicity, gestational age as linear and quadratic terms, season of birth, year, and random intercept for MSOA in addition to including the air pollutant or noise metrics shown above. IQR values for air pollutants: NO_2 (per IQR, 8.6 μg/m^3), NO_X (per IQR, 23.7 μg/m^3), $PM_{2.5\ traffic\ exhaust}$ (per IQR, 0.35 μg/m^3), $PM_{2.5\ traffic\ non\text{-}exhaust}$ (per IQR, 0.29 μg/m^3), $PM_{2.5}$ (per IQR, 2.2 μg/m^3), PM_{10} (per IQR, 3.0 μg/m^3), O_3 (per IQR, 8.4 μg/m^3)

Supplementary Table 1: Air pollution and road traffic noise exposures in the study population (N=540,365) and their correlations

		Air pollutants (pregnancy average) ($\mu g/m^3$)							Noise (annual average 2007) (dB)				
		NO_2	NO_X	$PM_{2.5\ traffic\text{-}exhaust}$	$PM_{2.5\ traffic\text{-}non\text{-}exhaust}$	$PM_{2.5}$	PM_{10}	O_3	$L_{Aeq,16hr}$	L_{night}	L_{day}	L_{eve}	L_{den}
**Summary statistics**	**Mean**	40.6	72.5	0.60	0.70	14.4	23.1	31.9	58.1	53.2	58.4	56.8	59.8
	**SD**	6.7	17.9	0.20	0.30	1.4	2.3	6.1	5.2	5.4	5.2	4.9	5.2
	**Min**	20.7	26.6	0.16	0.21	10.9	15.4	4.9	54.7	49.6	55.0	53.7	56.3
	**IQR**	8.6	23.7	0.35	0.29	2.2	3.0	8.4	3.5	3.9	3.6	3.1	3.7
	**Max**	185.2	520.3	7.10	6.60	26.6	48.8	51.4	86.0	80.0	86.4	84.4	86.7
**Spearman's correlation**	NO_2	1.00											
	NO_X	1.00	1.00										
	$PM_{2.5\ traffic\text{-}exhaust}$	0.95	0.95	1.00									
	$PM_{2.5\ traffic\text{-}non\text{-}exhaust}$	0.90	0.89	0.93	1.00								
	$PM_{2.5}$	0.68	0.69	0.68	0.45	1.00							
	PM_{10}	0.77	0.78	0.78	0.58	0.95	1.00						
	O_3	-0.76	-0.77	-0.68	-0.66	-0.46	-0.54	1.00					
	$L_{Aeq,\ 16hr}$	0.31	0.29	0.38	0.48	0.15	0.23	-0.14	1.00				
	L_{night}	0.33	0.32	0.41	0.50	0.16	0.24	-0.15	1.00	1.00			
	L_{day}	0.31	0.29	0.38	0.47	0.15	0.23	-0.14	1.00	1.00	1.00		
	L_{eve}	0.31	0.30	0.38	0.48	0.15	0.23	-0.14	1.00	1.00	1.00	1.00	
	L_{den}	0.33	0.31	0.40	0.50	0.15	0.24	-0.15	1.00	1.00	1.00	1.00	1.00

Supplementary Table 2: OR (95%CI) for term LBW and term SGA and mean difference (95% CI) in term birth weight (g) associated with potential confounding factors (univariate linear/logistic regression) in the study population (n=540,365)

	Term LBW			Term SGA			Term birth weight (g)		
**Variable**	**N cases**	**OR (95% CI)**	**p-value**	**N cases**	**OR (95% CI)**	**p-value**	**N**	**MD (95%CI)**	**p-value**
**Gestational Age (per completed week)**	13804	0.41 (0.40, 0.41)	<0.001				540365	136.38 (135.46, 137.31)	<0.001
**Tobacco expenditure (per £1) (COA)**	13804	1.08 (1.07, 1.08)	<0.001	44966	1.05 (1.04, 1.05)	<0.001	540365	-14.43 (-15.07, -13.79)	<0.001
**Sex**									
Male	5641	Reference					275546	Reference	
Female	8163	1.52 (1.47, 1.57)					264819	-125.50 (-127.99, -123.00)	
**Maternal age**									
<25 years	3339	Reference		11029	Reference		100931	Reference	
25-29 years	3888	0.83 (0.79, 0.87)		11937	0.74 (0.72, 0.76)		140353	52.70 (48.90, 56.49)	
30-34 years	3689	0.65 (0.62, 0.68)		12770	0.64 (0.63, 0.66)		169559	105.06 (101.40, 108.72)	
≥35 years	2888	0.67 (0.63, 0.70)		9230	0.61 (0.59, 0.63)		129522	121.26 (117.40, 125.13)	
p for trend							540365		
**Ethnicity**									
White	4890	Reference					286192	Reference	
Asian	4779	3.10 (2.97, 3.22)					93555	-274.77 (-278.17, -271.37)	
Black	2536	1.64 (1.56, 1.72)					91740	-111.47 (-114.89, -108.04)	
Other	1599	1.37 (1.29, 1.45)					68878	-91.38 (-95.21, -87.55)	
**Birth in marriage**									
Within marriage†	8617	Reference		25965	Reference		348157	Reference	
Sole registration	1234	1.40 (1.32, 1.49)		3971	1.60 (1.55, 1.66)		35937	-67.18 (-72.29, -62.07)	
Joint/same addr.	2316	0.89 (0.85, 0.93)		9503	1.24 (1.21, 1.27)		105239	28.08 (24.84, 31.33)	
Joint/different addr.	1637	1.31 (1.24, 1.38)		5527	1.59 (1.55, 1.64)		51032	-57.24 (-61.61, -52.86)	
**Year**									
2006	2839	Reference		9182	Reference		101770	Reference	
2007	2740	0.92 (0.87, 0.97)		8954	0.93 (0.90, 0.96)		106528	5.91 (1.86, 9.96)	
2008	2755	0.92 (0.88, 0.97)		8738	0.90 (0.87, 0.93)		106678	11.91 (7.86, 15.96)	
2009	2747	0.89 (0.85, 0.94)		8905	0.89 (0.86, 0.91)		110014	14.44 (10.42, 18.46)	
2010	2723	0.84 (0.80, 0.89)		9187	0.87 (0.84, 0.90)		115375	15.82 (11.85, 19.79)	
p for trend			<0.001			<0.001	540365		<0.001
**Season**									
Winter	3455	Reference		11058	Reference		130033	Reference	
Spring	3449	0.97 (0.93, 1.02)		11318	0.99 (0.97, 1.02)		133395	8.35 (4.75, 11.95)	
Summer	3463	0.94 (0.90, 0.99)		11325	0.96 (0.93, 0.99)		138418	17.04 (13.48, 20.61)	
Autumn	3437	0.93 (0.89, 0.98)		11265	0.95 (0.92, 0.98)		138519	16.31 (12.74, 19.88)	
**Carstairs quintile (COA)**									
1 - least deprived	1324	Reference		5961	Reference		85358	Reference	
2	1875	1.32 (1.23, 1.41)		7229	1.17 (1.13, 1.21)		92264	-34.31 (-38.68, -29.94)	
3	2448	1.58 (1.47, 1.69)		8217	1.25 (1.20, 1.29)		100934	-67.61 (-71.89, -63.34)	
4	3450	1.89 (1.77, 2.02)		10330	1.35 (1.31, 1.40)		119239	-99.05 (-103.17, -94.92)	
5 - most deprived	4707	2.17 (2.04, 2.30)		13229	1.43 (1.39, 1.48)		142570	-132.64 (-136.62, -128.66)	
p for trend			<0.001			<0.001	540365		<0.001
**Inner/Outer London**									
Inner	4372	Reference		14886	Reference		173181	Reference	
Outer	9432	1.02 (0.98, 1.06)		30080	0.93 (0.92, 0.95)		367184	-3.75 (-6.45, -1.06)	

Abbreviations: LBW, low birth weight (<2500g); SGA, small-for-gestational-age. †includes civil partnerships.

Supplementary Table 3: Mean difference (95% CI) in exposure metric associated with potential confounding factors (univariate linear regression) in the study population (n=540,365)

	NO_2 (pregnancy average, µg/m^3)			NO_X (pregnancy average, µg/m^3)		$PM_{2.5\ traffic\text{-}exhaust}$ (pregnancy average, µg/m^3)		$PM_{2.5\ traffic\text{-}non\text{-}exhaust}$ (pregnancy average, µg/m^3)	
Variable	**N**	**MD (95%CI)**	**p-value**	**MD (95%CI)**	**p-value**	**MD (95%CI)**	**p-value**	**MD (95%CI)**	**p-value**
**Gestational Age (per completed week)**	540365	0.06 (0.04, 0.07)		0.15 (0.12, 0.19)		0.00 (0.00, 0.00)		0.00 (0.00, 0.00)	
**Tobacco expenditure (per £1) (COA)**	540365	0.79 (0.78, 0.79)		2.03 (2.01, 2.05)		0.03 (0.03, 0.03)		0.03 (0.03, 0.03)	
**Sex**									
Male	275546	Reference		Reference		Reference		Reference	
Female	264819	-0.02 (-0.05, 0.02)		-0.05 (-0.14, 0.05)		-0.00 (-0.00, 0.00)		-0.00 (-0.00, 0.00)	
**Maternal age**									
<25 years	100931	Reference		Reference		Reference		Reference	
25-29 years	140353	-0.26 (-0.32, -0.21)		-0.70 (-0.84, -0.55)		-0.01 (-0.01, -0.01)		-0.01 (-0.01, -0.01)	
30-34 years	169559	-0.34 (-0.39, -0.29)		-0.86 (-1.00, -0.72)		-0.02 (-0.02, -0.01)		-0.01 (-0.02, -0.01)	
≥35 years	129522	-0.14 (-0.19, -0.08)		-0.29 (-0.44, -0.15)		-0.01 (-0.01, -0.01)		-0.01 (-0.01, -0.01)	
p for trend	540365		<0.001		<0.001		<0.001		<0.001
**Ethnicity**									
White	286192	Reference		Reference		Reference		Reference	
Asian	93555	1.05 (1.00, 1.10)		2.55 (2.42, 2.68)		0.03 (0.03, 0.03)		0.04 (0.04, 0.04)	
Black	91740	2.23 (2.18, 2.28)		5.67 (5.54, 5.80)		0.07 (0.07, 0.07)		0.08 (0.08, 0.08)	
Other	68878	1.58 (1.52, 1.63)		4.02 (3.88, 4.17)		0.05 (0.05, 0.06)		0.06 (0.06, 0.06)	
**Birth in marriage**									
Within marriage†	348157	Reference		Reference		Reference		Reference	
Sole registration	35937	0.71 (0.63, 0.78)		1.82 (1.62, 2.01)		0.03 (0.03, 0.03)		0.03 (0.02, 0.03)	
Joint/same address	105239	-0.66 (-0.70, -0.61)		-1.70 (-1.82, -1.58)		-0.02 (-0.02, -0.02)		-0.02 (-0.02, -0.02)	
Joint/different address	51032	0.20 (0.14, 0.27)		0.50 (0.34, 0.67)		0.01 (0.01, 0.01)		0.01 (0.01, 0.01)	
**Year**									
2006	101770	Reference		Reference		Reference		Reference	
2007	106528	-1.74 (-1.80, -1.69)		-5.84 (-5.98, -5.69)		-0.09 (-0.09, -0.09)		-0.02 (-0.03, -0.02)	
2008	106678	-0.23 (-0.29, -0.18)		0.17 (0.02, 0.31)		-0.08 (-0.08, -0.08)		0.05 (0.05, 0.05)	
2009	110014	-1.25 (-1.31, -1.20)		-4.51 (-4.65, -4.36)		-0.12 (-0.12, -0.12)		0.06 (0.06, 0.06)	
2010	115375	-5.04 (-5.10, -4.99)		-14.39 (-14.53, -14.24)		-0.21 (-0.21, -0.21)		-0.03 (-0.03, -0.02)	
p for trend	540365		<0.001		<0.001		<0.001		<0.001
**Season**									
Winter	130033	Reference		Reference		Reference		Reference	
Spring	133395	3.69 (3.64, 3.73)		9.48 (9.35, 9.61)		0.06 (0.06, 0.07)		0.07 (0.07, 0.07)	
Summer	138418	2.33 (2.29, 2.38)		6.06 (5.94, 6.19)		0.02 (0.02, 0.02)		0.03 (0.03, 0.04)	
Autumn	138519	-2.26 (-2.31, -2.21)		-6.18 (-6.31, -6.05)		-0.07 (-0.08, -0.07)		-0.06 (-0.06, -0.06)	
**Carstairs quintile (COA)**									
1 - least deprived	85358	Reference		Reference		Reference		Reference	
2	92264	2.05 (1.99, 2.11)		5.19 (5.03, 5.35)		0.07 (0.07, 0.07)		0.08 (0.07, 0.08)	
3	100934	3.10 (3.04, 3.16)		7.82 (7.67, 7.98)		0.10 (0.10, 0.10)		0.11 (0.11, 0.11)	
4	119239	3.84 (3.78, 3.89)		9.66 (9.50, 9.81)		0.12 (0.12, 0.12)		0.14 (0.13, 0.14)	
5 - most deprived	142570	5.69 (5.63, 5.74)		14.48 (14.33, 14.63)		0.19 (0.19, 0.19)		0.20 (0.20, 0.20)	
p for trend	540365		<0.001		<0.001		<0.001		<0.001
**Inner/Outer London**									
Inner	173181	Reference		Reference		Reference		Reference	
Outer	367184	-6.72 (-6.75, -6.69)		-17.46 (-17.55, -17.36)		-0.25 (-0.25, -0.24)		-0.23 (-0.23, -0.22)	

Supplementary Table 3 cont/d.

	$PM_{2.5}$ (pregnancy average, µg/m^3)		PM_{10} (pregnancy average, µg/m^3)		O_3 (pregnancy average, µg/m^3)		$L_{Aeq,16hr}$ (dB)		L_{night} (dB)	
Variable	**MD (95%CI)**	**p-value**	**MD (95%CI)**	**p-value**	**MD (95%CI)**	**p-value**	**MD (95%CI)**	**p-value**	**MD (95%CI)**	**p-value**
**Gestational Age (per completed week)**	0.02 (0.02, 0.02)		0.03 (0.03, 0.04)		-0.05 (-0.06, -0.04)		0.02 (0.01, 0.03)		0.02 (0.01, 0.03)	
**Tobacco expenditure (per £1) (COA)**	0.08 (0.08, 0.08)		0.17 (0.16, 0.17)		-0.42 (-0.43, -0.41)		0.15 (0.14, 0.15)		0.18 (0.17, 0.19)	
**Sex**										
Male	Reference		Reference		Reference		Reference		Reference	
Female	-0.01 (-0.01, 0.00)		-0.01 (-0.02, 0.01)		0.00 (-0.03, 0.04)		0.01 (-0.02, 0.04)		0.01 (-0.02, 0.04)	
**Maternal age**										
<25 years	Reference		Reference		Reference		Reference		Reference	
25-29 years	-0.07 (-0.09, -0.06)		-0.13 (-0.15, -0.11)		0.19 (0.14, 0.24)		-0.08 (-0.12, -0.04)		-0.10 (-0.14, -0.05)	
30-34 years	-0.10 (-0.11, -0.09)		-0.19 (-0.21, -0.18)		0.27 (0.22, 0.31)		-0.39 (-0.43, -0.35)		-0.41 (-0.45, -0.37)	
≥35 years	-0.09 (-0.10, -0.08)		-0.17 (-0.18, -0.15)		0.10 (0.05, 0.15)		-0.60 (-0.65, -0.56)		-0.63 (-0.67, -0.58)	
p for trend		<0.001		<0.001		<0.001		<0.001		<0.001
**Ethnicity**										
White	Reference		Reference		Reference		Reference		Reference	
Asian	0.10 (0.09, 0.11)		0.20 (0.18, 0.22)		-0.65 (-0.70, -0.61)		0.35 (0.31, 0.38)		0.36 (0.33, 0.40)	
Black	0.26 (0.25, 0.27)		0.52 (0.50, 0.53)		-1.32 (-1.36, -1.27)		0.45 (0.41, 0.49)		0.53 (0.49, 0.57)	
Other	0.16 (0.15, 0.17)		0.33 (0.31, 0.35)		-0.81 (-0.86, -0.76)		0.63 (0.58, 0.67)		0.67 (0.62, 0.71)	
**Birth in marriage**										
Within marriage[†]	Reference		Reference		Reference		Reference		Reference	
Sole registration	0.13 (0.11, 0.14)		0.24 (0.22, 0.27)		-0.42 (-0.48, -0.35)		0.39 (0.33, 0.44)		0.43 (0.37, 0.48)	
Joint/same address	-0.07 (-0.08, -0.06)		-0.12 (-0.14, -0.11)		0.42 (0.38, 0.46)		0.19 (0.15, 0.22)		0.19 (0.15, 0.22)	
Joint/different address	0.01 (-0.01, 0.02)		0.03 (0.01, 0.05)		-0.09 (-0.15, -0.04)		0.15 (0.10, 0.19)		0.18 (0.13, 0.23)	
**Year**										
2006	Reference		Reference		Reference		Reference		Reference	
2007	-1.27 (-1.28, -1.27)		-0.97 (-0.99, -0.96)		3.79 (3.75, 3.84)		0.04 (-0.00, 0.09)		0.04 (-0.00, 0.09)	
2008	-1.56 (-1.57, -1.56)		-1.56 (-1.58, -1.55)		0.21 (0.16, 0.25)		0.07 (0.03, 0.12)		0.07 (0.03, 0.12)	
2009	-2.11 (-2.11, -2.10)		-2.44 (-2.45, -2.43)		-2.97 (-3.01, -2.92)		0.11 (0.06, 0.15)		0.11 (0.06, 0.16)	
2010	-3.19 (-3.20, -3.18)		-4.58 (-4.59, -4.56)		6.08 (6.04, 6.12)		0.19 (0.15, 0.24)		0.20 (0.16, 0.25)	
p for trend		<0.001		<0.001		<0.001		<0.001		<0.001
**Season**										
Winter	Reference		Reference		Reference		Reference		Reference	
Spring	0.88 (0.87, 0.89)		1.20 (1.19, 1.22)		-3.66 (-3.70, -3.62)		-0.07 (-0.11, -0.03)		-0.08 (-0.12, -0.04)	
Summer	1.09 (1.08, 1.10)		1.18 (1.16, 1.20)		1.80 (1.76, 1.83)		-0.03 (-0.07, 0.01)		-0.04 (-0.08, 0.00)	
Autumn	-0.02 (-0.03, -0.01)		-0.33 (-0.34, -0.31)		5.04 (5.00, 5.08)		-0.02 (-0.06, 0.02)		-0.02 (-0.06, 0.02)	
**Carstairs quintile (COA)**										
1 - least deprived	Reference		Reference		Reference		Reference		Reference	
2	0.17 (0.15, 0.18)		0.37 (0.35, 0.39)		-1.09 (-1.15, -1.04)		0.83 (0.79, 0.88)		0.90 (0.85, 0.95)	
3	0.26 (0.24, 0.27)		0.56 (0.54, 0.58)		-1.74 (-1.79, -1.68)		1.07 (1.03, 1.12)		1.17 (1.12, 1.22)	
4	0.34 (0.33, 0.35)		0.75 (0.73, 0.77)		-2.16 (-2.21, -2.11)		1.18 (1.14, 1.23)		1.31 (1.26, 1.35)	
5 - most deprived	0.56 (0.55, 0.57)		1.18 (1.16, 1.20)		-3.18 (-3.23, -3.13)		1.21 (1.16, 1.25)		1.42 (1.37, 1.46)	
p for trend		<0.001		<0.001		<0.001		<0.001		<0.001
**Inner/Outer London**										
Inner	Reference		Reference		Reference		Reference		Reference	
Outer	-0.63 (-0.63, -0.62)		-1.29 (-1.30, -1.28)		3.68 (3.64, 3.71)		-0.80 (-0.83, -0.77)		-1.04 (-1.07, -1.01)	

[†]includes civil partnerships.

Supplementary Table 4: Odds of term LBW (<2500g and ≥37 weeks gestation) associated with air pollutants and noise (OR, 95% confidence intervals)

	Unadjusted single-exposure model Term LBW				Adjusted single-exposure model Term LBW	
**Exposure**	**N**	**N cases**	**OR (95% CI)**	**p-value**	**OR (95% CI)**	**p-value**
**NO_2 (per IQR)**	540,365	13,804	1.10 (1.07, 1.12)		1.03 (1.00, 1.06)	
**NO_X (per IQR)**	540,365	13,804	1.09 (1.07, 1.12)		1.03 (1.01, 1.06)	
**$PM_{2.5\ traffic\text{-}exhaust}$ (per IQR)**	540,365	13,804	1.10 (1.08, 1.13)		1.04 (1.01, 1.07)	
**$PM_{2.5\ traffic\text{-}non\text{-}exhaust}$ (per IQR)**	540,365	13,804	1.07 (1.05, 1.09)		1.02 (1.00, 1.04)	
**$PM_{2.5}$ (per IQR)**	540,365	13,804	1.10 (1.08, 1.13)		1.06 (1.01, 1.12)	
**PM_{10} (per IQR)**	540,365	13,804	1.09 (1.07, 1.12)		1.03 (0.99, 1.07)	
**O_3 (per IQR)**	540,365	13,804	0.92 (0.90, 0.95)		0.96 (0.93, 0.99)	
**Day-time noise, $L_{Aeq,16hr}$**						
<55dB	157,491	3,956	Reference		Reference	
55-<60 dB	265,603	6,719	1.01 (0.97, 1.05)		0.98 (0.94, 1.02)	
60-<65 dB	40,755	1,093	1.07 (1.00, 1.14)		1.02 (0.95, 1.10)	
≥65dB	76,516	2,036	1.06 (1.00, 1.12)		1.01 (0.95, 1.07)	
p for trend	540,365			0.012		0.572
**Night-time noise, L_{night}**						
<50dB	162,260	4,032	Reference		Reference	
50-<55 dB	257,045	6,546	1.03 (0.99, 1.07)		0.99 (0.95, 1.03)	
55-<60 dB	40,256	1,054	1.06 (0.99, 1.13)		1.01 (0.94, 1.09)	
60-<65 dB	46,994	1,253	1.07 (1.01, 1.15)		1.02 (0.95, 1.09)	
≥65dB	33,810	919	1.10 (1.02, 1.18)		1.03 (0.95, 1.11)	
p for trend	540,365			0.002		0.349

Adjusted model covariates: sex, maternal age, birth registration type, tobacco expenditure (COA-level), Carstairs quintile (COA-level), individual-level ethnicity, gestational age as linear and quadratic terms, season of birth, year (linear term) and random intercept for MSOA.
IQR values for air pollutants: NO_2 (per IQR, 8.6 μg/m^3), NO_X (per IQR, 23.7 μg/m^3), $PM_{2.5\ traffic\ exhaust}$ (per IQR, 0.35 μg/m^3), $PM_{2.5\ traffic\ non\text{-}exhaust}$ (per IQR, 0.29 μg/m^3), $PM_{2.5}$ (per IQR, 2.2 μg/m^3), PM_{10} (per IQR, 3.0 μg/m^3), O_3 (per IQR, 8.4 μg/m^3)

Supplementary Table 5: Odds of term small-for-gestational-age (SGA) associated with air pollutants and noise (odds ratios with 95% confidence intervals)

	Unadjusted single-exposure model Term sex-ethnic-specific SGA				Adjusted single-exposure model Term sex-ethnic-specific SGA	
**Exposure**	**N**	**N cases**	**OR (95% CI)**	**p-value**	**OR (95% CI)**	**p-value**
**NO_2 (per IQR)**	471489	44966	1.05 (1.04, 1.07)		1.01 (1.00, 1.03)	
**NO_X (per IQR)**	471489	44966	1.05 (1.04, 1.07)		1.01 (1.00, 1.03)	
**$PM_{2.5\ traffic\text{-}exhaust}$ (per IQR)**	471489	44966	1.07 (1.06, 1.08)		1.02 (1.01, 1.04)	
**$PM_{2.5\ traffic\text{-}non\text{-}exhaust}$ (per IQR)**	471489	44966	1.03 (1.02, 1.05)		1.01 (1.00, 1.02)	
**$PM_{2.5}$ (per IQR)**	471489	44966	1.08 (1.07, 1.10)		1.03 (1.00, 1.06)	
**PM_{10} (per IQR)**	471489	44966	1.07 (1.05, 1.08)		1.01 (0.99, 1.03)	
**O_3 (per IQR)**	471489	44966	0.96 (0.94, 0.97)		0.99 (0.97, 1.01)	
**Day-time noise, $L_{Aeq,16hr}$**						
<55dB	138696	13113	Reference		Reference	
55-<60 dB	232346	22008	1.00 (0.98, 1.03)		0.99 (0.97, 1.01)	
60-<65 dB	35334	3458	1.04 (1.00, 1.08)		1.02 (0.98, 1.06)	
≥65dB	65113	6387	1.04 (1.01, 1.07)		1.00 (0.97, 1.04)	
p for trend	471489			0.004		0.567
**Night-time noise, L_{night}**						
<50dB	142880	13406	Reference		Reference	
50-<55 dB	224864	21351	1.01 (0.99, 1.04)		1.00 (0.98, 1.02)	
55-<60 dB	34960	3433	1.05 (1.01, 1.09)		1.03 (0.99, 1.07)	
60-<65 dB	40344	3899	1.03 (1.00, 1.07)		1.00 (0.96, 1.04)	
≥65dB	28441	2877	1.09 (1.04, 1.13)		1.03 (0.99, 1.08)	
p for trend	471489			<0.001		0.156

Adjusted model covariates: maternal age, birth registration type, tobacco expenditure (COA-level), Carstairs quintile (COA-level), season of birth, year (linear term) and random intercept for MSOA. IQR values for air pollutants: NO_2 (per IQR, 8.6 μg/m^3), NO_X (per IQR, 23.7 μg/m^3), $PM_{2.5\ traffic\ exhaust}$ (per IQR, 0.35 μg/m^3), $PM_{2.5\ traffic\ non\text{-}exhaust}$ (per IQR, 0.29 μg/m^3), $PM_{2.5}$ (per IQR, 2.2 μg/m^3), PM_{10} (per IQR, 3.0 μg/m^3), O_3 (per IQR, 8.4 μg/m^3)

Supplementary Table 6: Mean difference in term birth weight (g) associated with air pollutants and noise (with 95% confidence intervals)

		Unadjusted single-exposure model Term birth weight (g)		Adjusted single-exposure model Term birth weight (g)	
**Exposure**	**N**	**MD (95%CI)**	**p-value**	**MD (95%CI)**	**p-value**
**NO_2 (per IQR)**	540,365	-20.67 (-22.30, -19.04)		-10.97 (-12.98, -8.96)	
**NO_X (per IQR)**	540,365	-20.15 (-21.81, -18.49)		-10.74 (-12.76, -8.73)	
**$PM_{2.5\ traffic\text{-}exhaust}$ (per IQR)**	540,365	-23.09 (-24.89, -21.29)		-12.43 (-14.51, -10.35)	
**$PM_{2.5\ traffic\text{-}non\text{-}exhaust}$ (per IQR)**	540,365	-17.24 (-18.68, -15.80)		-7.41 (-8.96, -5.86)	
**$PM_{2.5}$ (per IQR)**	540,365	-14.61 (-16.58, -12.65)		-12.94 (-16.41, -9.47)	
**PM_{10} (per IQR)**	540,365	-14.38 (-16.08, -12.69)		-7.27 (-9.84, -4.70)	
**O_3 (per IQR)**	540,365	14.53 (12.81, 16.25)		5.12 (3.11, 7.12)	
**Day-time noise, $L_{Aeq,16hr}$**					
<55dB	157,491	Reference		Reference	
55-<60 dB	265,603	-3.80 (-6.74, -0.87)		2.02 (-0.73, 4.78)	
60-<65 dB	40,755	-14.63 (-19.76, -9.49)		-4.70 (-9.39, -0.02)	
≥65dB	76,516	-19.88 (-23.95, -15.81)		-5.23 (-9.00, -1.45)	
p for trend	540,365		<0.001		0.001
**Night-time noise, L_{night}**					
<50dB	162,260	Reference		Reference	
50-<55 dB	257,045	-6.63 (-9.56, -3.70)		0.08 (-2.67, 2.83)	
55-<60 dB	40,256	-14.62 (-19.76, -9.47)		-4.64 (-9.33, 0.06)	
60-<65 dB	46,994	-17.33 (-22.17, -12.49)		-4.34 (-8.76, 0.09)	
≥65dB	33,810	-31.65 (-37.17, -26.13)		-12.66 (-17.77, -7.55)	
p for trend	540,365		<0.001		<0.001

Adjusted model covariates: sex, maternal age, birth registration type, tobacco expenditure (COA-level), Carstairs quintile (COA-level), individual-level ethnicity, gestational age as linear and quadratic terms, season of birth, year (linear term) and random intercept for MSOA.
IQR values for air pollutants: NO_2 (per IQR, 8.6 μg/m^3), NO_X (per IQR, 23.7 μg/m^3), $PM_{2.5\ traffic\ exhaust}$ (per IQR, 0.35 μg/m^3), $PM_{2.5\ traffic\ non\text{-}exhaust}$ (per IQR, 0.29 μg/m^3), $PM_{2.5}$ (per IQR, 2.2 μg/m^3), PM_{10} (per IQR, 3.0 μg/m^3), O_3 (per IQR, 8.4 μg/m^3)

Supplementary Table 7: Joint air-pollutant-noise models: Mean difference (95% CI) in birth weight (g) associated with air pollutants and noise amongst term births

	Joint air pollutant-noise model* Term birth weight (g)		
**Exposure**	**N**	**MD (95%CI)**	**p-value**
**Adjusted for night-time noise**			
**NO_2 (per IQR)**	540,365	-10.43 (-12.64, -8.22)	
**NO_X (per IQR)**	540,365	-10.07 (-12.26, -7.88)	
**$PM_{2.5\ traffic\text{-}exhaust}$ (per IQR)**	540,365	-12.54 (-14.92, -10.16)	
**$PM_{2.5\ traffic\text{-}non\text{-}exhaust}$ (per IQR)**	540,365	-7.27 (-9.14, -5.41)	
**$PM_{2.5}$ (per IQR)**	540,365	-11.02 (-14.67, -7.37)	
**PM_{10} (per IQR)**	540,365	-5.31 (-8.09, -2.52)	
**O_3 (per IQR)**	540,365	4.41 (2.38, 6.44)	
**Adjusted for NO_2**			
**Night-time noise, L_{night}**			
<50dB	162260	Reference	
50-<55 dB	257045	1.79 (-0.97, 4.56)	
55-<60 dB	40256	-1.57 (-6.30, 3.16)	
60-<65 dB	46994	0.07 (-4.45, 4.60)	
≥65dB	33810	-2.71 (-8.25, 2.83)	
p for trend	540365		0.330
**Adjusted for day-time noise**			
**NO_2 (per IQR)**	540,365	-11.06 (-13.22, -8.91)	
**NO_X (per IQR)**	540,365	-10.70 (-12.84, -8.56)	
**$PM_{2.5\ traffic\text{-}exhaust}$ (per IQR)**	540,365	-13.14 (-15.44, -10.84)	
**$PM_{2.5\ traffic\text{-}non\text{-}exhaust}$ (per IQR)**	540,365	-8.01 (-9.82, -6.21)	
**$PM_{2.5}$ (per IQR)**	540,365	-12.06 (-15.68, -8.45)	
**PM_{10} (per IQR)**	540,365	-6.33 (-9.07, -3.60)	
**O_3 (per IQR)**	540,365	4.74 (2.72, 6.76)	
**Adjusted for NO_2**			
**Day-time noise, $L_{Aeq,16hr}$**			
<55dB	157491	Reference	
55-<60 dB	265603	3.61 (0.85, 6.38)	
60-<65 dB	40755	-1.53 (-6.25, 3.19)	
≥65dB	76516	1.82 (-2.21, 5.84)	
p for trend	540365		0.715

*Models are adjusted as follow: sex, maternal age, birth registration type, tobacco expenditure (COA-level), Carstairs quintile (COA-level), individual-level ethnicity, gestational age as linear and quadratic terms, season of birth, year (linear term) and random intercept for MSOA. All air pollution estimates are adjusted for either night-time (L_{night}) or daytime noise ($L_{Aeq,16hr}$) as specified in the table, and noise estimates are adjusted for traffic-related air pollution exposure (NO2). IQR values for air pollutants: NO_2 (per IQR, 8.6 $\mu g/m^3$), NO_X (per IQR, 23.7 $\mu g/m^3$), $PM_{2.5\ traffic\ exhaust}$ (per IQR, 0.35 $\mu g/m^3$), $PM_{2.5\ traffic\ non\text{-}exhaust}$ (per IQR, 0.29 $\mu g/m^3$), $PM_{2.5}$ (per IQR, 2.2 $\mu g/m^3$), PM_{10} (per IQR, 3.0 $\mu g/m^3$), O_3 (per IQR, 8.4 $\mu g/m^3$)

Supplementary Table 8: Associations between noise on the continuous scale (per IQR) and odds of LBW and SGA and mean difference in birth weight (g) amongst term births, in unadjusted, adjusted and joint air pollutant-noise models

			Term LBW		
**Model**	**N**	**Noise**	**OR (95% CI)**	**Air pollutant**	**OR (95% CI)**
**Unadjusted**	540365	**L_{night} (per IQR)**	1.07 (1.05, 1.09)		
**Adjusted***	540365	**L_{night} (per IQR)**	1.01 (0.99, 1.02)		
**Joint air pollutant-noise model***	540365	**L_{night} (per IQR)**	1.00 (0.99, 1.01)	**NO_2 (per IQR)**	1.03 (1.00, 1.06)
**Joint air pollutant-noise model***	540365	**L_{night} (per IQR)**	1.00 (0.99, 1.01)	**NO_X (per IQR)**	1.03 (1.00, 1.06)
**Joint air pollutant-noise model***	540365	**L_{night} (per IQR)**	1.00 (0.98, 1.01)	**$PM_{2.5\ traffic\text{-}exhaust}$ (per IQR)**	1.04 (1.01, 1.08)
**Joint air pollutant-noise model***	540365	**L_{night} (per IQR)**	1.00 (0.98, 1.01)	**$PM_{2.5\ traffic\text{-}non\text{-}exhaust}$ (per IQR)**	1.02 (1.00, 1.05)
**Joint air pollutant-noise model***	540365	**L_{night} (per IQR)**	1.00 (0.99, 1.02)	**$PM_{2.5}$ (per IQR)**	1.06 (1.01, 1.12)
**Joint air pollutant-noise model***	540365	**L_{night} (per IQR)**	1.00 (0.99, 1.02)	**PM_{10} (per IQR)**	1.03 (0.99, 1.07)
**Joint air pollutant-noise model***	540365	**L_{night} (per IQR)**	1.00 (0.99, 1.02)	**O_3 (per IQR)**	0.96 (0.94, 0.99)
**Unadjusted**	540365	**$L_{Aeq,16hr}$ (per IQR)**	1.10 (1.08, 1.13)		
**Adjusted***	540365	**$L_{Aeq,16hr}$ (per IQR)**	1.01 (0.99, 1.02)		
**Joint air pollutant-noise model***	540365	**$L_{Aeq,16hr}$ (per IQR)**	1.00 (0.99, 1.01)	**NO_2 (per IQR)**	1.03 (1.00, 1.06)
**Joint air pollutant-noise model***	540365	**$L_{Aeq,16hr}$ (per IQR)**	1.00 (0.99, 1.01)	**NO_X (per IQR)**	1.03 (1.00, 1.06)
**Joint air pollutant-noise model***	540365	**$L_{Aeq,16hr}$ (per IQR)**	1.00 (0.98, 1.01)	**$PM_{2.5\ traffic\text{-}exhaust}$ (per IQR)**	1.04 (1.01, 1.08)
**Joint air pollutant-noise model***	540365	**$L_{Aeq,16hr}$ (per IQR)**	1.00 (0.99, 1.01)	**$PM_{2.5\ traffic\text{-}non\text{-}exhaust}$ (per IQR)**	1.02 (1.00, 1.05)
**Joint air pollutant-noise model***	540365	**$L_{Aeq,16hr}$ (per IQR)**	1.00 (0.99, 1.01)	**$PM_{2.5}$ (per IQR)**	1.06 (1.01, 1.12)
**Joint air pollutant-noise model***	540365	**$L_{Aeq,16hr}$ (per IQR)**	1.00 (0.99, 1.02)	**PM_{10} (per IQR)**	1.03 (0.99, 1.07)
**Joint air pollutant-noise model***	540365	**$L_{Aeq,16hr}$ (per IQR)**	1.00 (0.99, 1.02)	**O_3 (per IQR)**	0.96 (0.94, 0.99)

			Term SGA		
**Model**	**N**	**Noise**	**OR (95% CI)**	**Air pollutant**	**OR (95% CI)**
**Unadjusted**	471489	**L_{night} (per IQR)**	1.01 (1.01, 1.02)		
**Adjusted***	471489	**L_{night} (per IQR)**	1.01 (1.00, 1.01)		
**Joint air pollutant-noise model***	471489	**L_{night} (per IQR)**	1.00 (1.00, 1.01)	**NO_2 (per IQR)**	1.01 (0.99, 1.03)
**Joint air pollutant-noise model***	471489	**L_{night} (per IQR)**	1.00 (1.00, 1.01)	**NO_X (per IQR)**	1.01 (1.00, 1.03)
**Joint air pollutant-noise model***	471489	**L_{night} (per IQR)**	1.00 (0.99, 1.01)	**$PM_{2.5\ traffic\text{-}exhaust}$ (per IQR)**	1.02 (1.00, 1.04)
**Joint air pollutant-noise model***	471489	**L_{night} (per IQR)**	1.00 (0.99, 1.01)	**$PM_{2.5\ traffic\text{-}non\text{-}exhaust}$ (per IQR)**	1.01 (0.99, 1.02)
**Joint air pollutant-noise model***	471489	**L_{night} (per IQR)**	1.00 (1.00, 1.01)	**$PM_{2.5}$ (per IQR)**	1.03 (1.00, 1.06)
**Joint air pollutant-noise model***	471489	**L_{night} (per IQR)**	1.00 (1.00, 1.01)	**PM_{10} (per IQR)**	1.00 (0.98, 1.03)
**Joint air pollutant-noise model***	471489	**L_{night} (per IQR)**	1.00 (1.00, 1.01)	**O_3 (per IQR)**	0.99 (0.98, 1.01)

			**Term SGA**		
**Model**	**N**	**Noise**	**OR (95% CI)**	**Air pollutant**	**OR (95% CI)**
**Unadjusted**	471489	**$L_{Aeq,16hr}$ (per IQR)**	1.01 (1.01, 1.02)		
**Adjusted***	471489	**$L_{Aeq,16hr}$ (per IQR)**	1.00 (1.00, 1.01)		
**Joint air pollutant-noise model***	471489	**$L_{Aeq,16hr}$ (per IQR)**	1.00 (1.00, 1.01)	**NO_2 (per IQR)**	1.01 (0.99, 1.03)
**Joint air pollutant-noise model***	471489	**$L_{Aeq,16hr}$ (per IQR)**	1.00 (1.00, 1.01)	**NO_X (per IQR)**	1.01 (1.00, 1.03)
**Joint air pollutant-noise model***	471489	**$L_{Aeq,16hr}$ (per IQR)**	1.00 (0.99, 1.01)	**$PM_{2.5\ traffic\text{-}exhaust}$ (per IQR)**	1.02 (1.00, 1.04)
**Joint air pollutant-noise model***	471489	**$L_{Aeq,16hr}$ (per IQR)**	1.00 (0.99, 1.01)	**$PM_{2.5\ traffic\text{-}non\text{-}exhaust}$ (per IQR)**	1.01 (0.99, 1.02)
**Joint air pollutant-noise model***	471489	**$L_{Aeq,16hr}$ (per IQR)**	1.00 (1.00, 1.01)	**$PM_{2.5}$ (per IQR)**	1.03 (1.00, 1.06)
**Joint air pollutant-noise model***	471489	**$L_{Aeq,16hr}$ (per IQR)**	1.00 (1.00, 1.01)	**PM_{10} (per IQR)**	1.00 (0.98, 1.03)
**Joint air pollutant-noise model***	471489	**$L_{Aeq,16hr}$ (per IQR)**	1.00 (1.00, 1.01)	**O_3 (per IQR)**	0.99 (0.98, 1.01)

			**Term birth weight (g)**		
**Model**	**N**	**Noise**	**MD (95%CI)**	**Air pollutant**	**MD (95%CI)**
**Unadjusted**	540365	**L_{night} (per IQR)**	-5.74 (-6.66, -4.83)		
**Adjusted***	540365	**L_{night} (per IQR)**	-2.24 (-3.08, -1.40)		
**Joint air pollutant-noise model***	540365	**L_{night} (per IQR)**	-0.54 (-1.46, 0.38)	**NO_2 (per IQR)**	-10.45 (-12.64, -8.25)
**Joint air pollutant-noise model***	540365	**L_{night} (per IQR)**	-0.69 (-1.60, 0.22)	**NO_X (per IQR)**	-10.10 (-12.28, -7.92)
**Joint air pollutant-noise model***	540365	**L_{night} (per IQR)**	0.15 (-0.81, 1.10)	**$PM_{2.5\ traffic\text{-}exhaust}$ (per IQR)**	-12.61 (-14.98, -10.24)
**Joint air pollutant-noise model***	540365	**L_{night} (per IQR)**	-0.04 (-1.05, 0.97)	**$PM_{2.5\ traffic\text{-}non\text{-}exhaust}$ (per IQR)**	-7.37 (-9.23, -5.50)
**Joint air pollutant-noise model***	540365	**L_{night} (per IQR)**	-1.45 (-2.33, -0.56)	**$PM_{2.5}$ (per IQR)**	-11.12 (-14.76, -7.47)
**Joint air pollutant-noise model***	540365	**L_{night} (per IQR)**	-1.59 (-2.50, -0.68)	**PM_{10} (per IQR)**	-5.41 (-8.19, -2.63)
**Joint air pollutant-noise model***	540365	**L_{night} (per IQR)**	-1.98 (-2.83, -1.13)	**O_3 (per IQR)**	4.43 (2.41, 6.46)
**Unadjusted**	540365	**$L_{Aeq,16hr}$ (per IQR)**	-5.13 (-5.98, -4.27)		
**Adjusted***	540365	**$L_{Aeq,16hr}$ (per IQR)**	-2.02 (-2.81, -1.23)		
**Joint air pollutant-noise model***	540365	**$L_{Aeq,16hr}$ (per IQR)**	-0.44 (-1.30, 0.41)	**NO_2 (per IQR)**	-10.52 (-12.71, -8.33)
**Joint air pollutant-noise model***	540365	**$L_{Aeq,16hr}$ (per IQR)**	-0.58 (-1.43, 0.27)	**NO_X (per IQR)**	-10.18 (-12.35, -8.00)
**Joint air pollutant-noise model***	540365	**$L_{Aeq,16hr}$ (per IQR)**	0.20 (-0.69, 1.09)	**$PM_{2.5\ traffic\text{-}exhaust}$ (per IQR)**	-12.68 (-15.04, -10.32)
**Joint air pollutant-noise model***	540365	**$L_{Aeq,16hr}$ (per IQR)**	0.05 (-0.90, 0.99)	**$PM_{2.5\ traffic\text{-}non\text{-}exhaust}$ (per IQR)**	-7.46 (-9.32, -5.61)
**Joint air pollutant-noise model***	540365	**$L_{Aeq,16hr}$ (per IQR)**	-1.28 (-2.11, -0.46)	**$PM_{2.5}$ (per IQR)**	-11.24 (-14.88, -7.60)
**Joint air pollutant-noise model***	540365	**$L_{Aeq,16hr}$ (per IQR)**	-1.40 (-2.25, -0.56)	**PM_{10} (per IQR)**	-5.54 (-8.31, -2.76)
**Joint air pollutant-noise model***	540365	**$L_{Aeq,16hr}$ (per IQR)**	-1.78 (-2.58, -0.98)	**O_3 (per IQR)**	4.48 (2.45, 6.50)

*Models are adjusted as follows. Term LBW: sex, maternal age, birth registration type, tobacco expenditure (COA-level), Carstairs quintile (COA-level), individual-level ethnicity, gestational age as linear and quadratic terms, season of birth, year (linear term) and random intercept for MSOA. Term SGA: maternal age, birth registration type, tobacco expenditure (COA-level), Carstairs quintile (COA-level), season of birth, year

(linear term) and random intercept for MSOA. Term birth weight: sex, maternal age, birth registration type, tobacco expenditure (COA-level), Carstairs quintile (COA-level), individual-level ethnicity, gestational age as linear and quadratic terms, season of birth, year (linear term) and random intercept for MSOA. **Joint air pollutant-noise models are additionally adjusted for the air pollutant stated in the table above. IQR values for noise and air pollutants: L_{night} (per IQR, 3.9 dB), $L_{Aeq,16hr}$ (per IQR, 3.5 dB), NO_2 (per IQR, 8.6 μg/m^3), NO_X (per IQR, 23.7 μg/m^3), $PM_{2.5\ traffic\ exhaust}$ (per IQR, 0.35 μg/m^3), $PM_{2.5\ traffic\ non\text{-}exhaust}$ (per IQR, 0.29 μg/m^3), $PM_{2.5}$ (per IQR, 2.2 μg/m^3), PM_{10} (per IQR, 3.0 μg/m^3), O_3 (per IQR, 8.4 μg/m^3).

Supplementary Table 9: Joint air-pollutant-noise models: Adjusted OR (95%CI) for LBW and SGA, and mean difference (95% CI) in birth weight (g) associated with noise amongst term births, after adjustment for each air pollutant

	Joint air pollutant-noise model* Term LBW			Joint air pollutant-noise model* Term SGA			Joint air pollutant-noise model* Term birth weight (g)		
**Exposure**	**N**	**OR (95% CI)**	**p-value**	**N**	**OR (95% CI)**	**p-value**	**N**	**MD (95%CI)**	**p-value**
**Adjusted for NO_2**									
**$L_{Aeq,16hr}$**									
<55dB	157491	Reference		138696	Reference		157491	Reference	
55-<60 dB	265603	0.97 (0.93, 1.02)		232346	0.99 (0.96, 1.01)		265603	3.61 (0.85, 6.38)	
60-<65 dB	40755	1.01 (0.94, 1.09)		35334	1.01 (0.97, 1.05)		40755	-1.53 (-6.25, 3.19)	
≥65dB	76516	0.98 (0.93, 1.05)		65113	0.99 (0.96, 1.03)		76516	1.82 (-2.21, 5.84)	
p for trend	540365		0.802	471489		0.957	540365		0.715
**L_{night}**									
<50dB	162260	Reference		142880	Reference		162260	Reference	
50-<55 dB	257045	0.98 (0.94, 1.03)		224864	1.00 (0.97, 1.02)		257045	1.79 (-0.97, 4.56)	
55-<60 dB	40256	1.00 (0.93, 1.07)		34960	1.02 (0.98, 1.06)		40256	-1.57 (-6.30, 3.16)	
60-<65 dB	46994	1.00 (0.94, 1.08)		40344	1.00 (0.96, 1.04)		46994	0.07 (-4.45, 4.60)	
≥65dB	33810	0.99 (0.91, 1.08)		28441	1.02 (0.97, 1.07)		33810	-2.71 (-8.25, 2.83)	
p for trend	540365		0.962	471489		0.432	540365		0.330
**Adjusted for NO_X**									
**$L_{Aeq,16hr}$**									
<55dB	157491	Reference		138696	Reference		157491	Reference	
55-<60 dB	265603	0.97 (0.93, 1.02)		232346	0.99 (0.97, 1.01)		265603	3.42 (0.66, 6.18)	
60-<65 dB	40755	1.01 (0.94, 1.09)		35334	1.01 (0.97, 1.05)		40755	-1.90 (-6.62, 2.81)	
≥65dB	76516	0.98 (0.93, 1.05)		65113	0.99 (0.96, 1.03)		76516	1.14 (-2.85, 5.13)	
p for trend	540365		0.800	471489		0.977	540365		0.976
**L_{night}**									
<50dB	162260	Reference		142880	Reference		162260	Reference	
50-<55 dB	257045	0.98 (0.94, 1.03)		224864	1.00 (0.97, 1.02)		257045	1.60 (-1.17, 4.36)	
55-<60 dB	40256	1.00 (0.93, 1.07)		34960	1.02 (0.98, 1.06)		40256	-1.92 (-6.65, 2.80)	
60-<65 dB	46994	1.01 (0.94, 1.08)		40344	1.00 (0.96, 1.04)		46994	-0.42 (-4.93, 4.08)	
≥65dB	33810	0.99 (0.91, 1.07)		28441	1.02 (0.97, 1.07)		33810	-3.56 (-9.05, 1.93)	
p for trend	540365		0.969	471489		0.421	540365		0.187

Exposure	Joint air pollutant-noise model* Term LBW N	OR (95% CI)	p-value	Joint air pollutant-noise model* Term SGA N	OR (95% CI)	p-value	Joint air pollutant-noise model* Term birth weight (g) N	MD (95%CI)	p-value
**Adjusted for $PM_{2.5\ traffic\text{-}exhaust}$**									
**$L_{Aeq,16hr}$**									
<55dB	157491	Reference		138696	Reference		157491	Reference	
55-<60 dB	265603	0.97 (0.93, 1.01)		232346	0.99 (0.96, 1.01)		265603	3.95 (1.18, 6.71)	
60-<65 dB	40755	1.01 (0.94, 1.08)		35334	1.01 (0.97, 1.05)		40755	-0.45 (-5.19, 4.29)	
≥65dB	76516	0.97 (0.91, 1.04)		65113	0.99 (0.95, 1.02)		76516	4.35 (0.21, 8.49)	
p for trend	540365		0.535	471489		0.581	540365		0.113
**L_{night}**									
<50dB	162260	Reference		142880	Reference		162260	Reference	
50-<55 dB	257045	0.98 (0.94, 1.02)		224864	0.99 (0.97, 1.02)		257045	2.15 (-0.62, 4.92)	
55-<60 dB	40256	0.99 (0.92, 1.07)		34960	1.02 (0.98, 1.06)		40256	-0.54 (-5.29, 4.21)	
60-<65 dB	46994	1.00 (0.93, 1.07)		40344	0.99 (0.95, 1.03)		46994	1.71 (-2.86, 6.29)	
≥65dB	33810	0.97 (0.89, 1.06)		28441	1.01 (0.96, 1.06)		33810	0.97 (-4.77, 6.71)	
p for trend	540365		0.715	471489		0.803	540365		0.726
**Adjusted for $PM_{2.5\ traffic\ non\text{-}exhaust}$**									
**$L_{Aeq,16hr}$**									
<55dB	157491	Reference		138696	Reference		157491	Reference	
55-<60 dB	265603	0.97 (0.93, 1.01)		232346	0.99 (0.96, 1.01)		265603	3.89 (1.11, 6.67)	
60-<65 dB	40755	1.01 (0.94, 1.08)		35334	1.01 (0.97, 1.05)		40755	-0.44 (-5.22, 4.34)	
≥65dB	76516	0.98 (0.92, 1.04)		65113	0.99 (0.96, 1.03)		76516	4.26 (-0.09, 8.60)	
p for trend	540365		0.639	471489		0.862	540365		0.135
**L_{night}**									
<50dB	162260	Reference		142880	Reference		162260	Reference	
50-<55 dB	257045	0.98 (0.94, 1.03)		224864	1.00 (0.97, 1.02)		257045	1.93 (-0.86, 4.72)	
55-<60 dB	40256	1.00 (0.93, 1.07)		34960	1.02 (0.98, 1.07)		40256	-0.79 (-5.58, 4.01)	
60-<65 dB	46994	1.00 (0.93, 1.07)		40344	1.00 (0.96, 1.04)		46994	1.81 (-2.90, 6.51)	
≥65dB	33810	0.98 (0.90, 1.08)		28441	1.02 (0.97, 1.08)		33810	-0.65 (-6.64, 5.34)	
p for trend	540365		0.883	471489		0.454	540365		0.965

Exposure	N	Joint air pollutant-noise model* Term LBW OR (95% CI)	p-value	N	Joint air pollutant-noise model* Term SGA OR (95% CI)	p-value	N	Joint air pollutant-noise model* Term birth weight (g) MD (95%CI)	p-value
**Adjusted for $PM_{2.5}$**									
**$L_{Aeq,16hr}$**									
<55dB	157491	Reference		138696	Reference		157491	Reference	
55-<60 dB	265603	0.97 (0.94, 1.02)		232346	0.99 (0.97, 1.01)		265603	2.75 (-0.01, 5.50)	
60-<65 dB	40755	1.01 (0.94, 1.09)		35334	1.01 (0.97, 1.05)		40755	-3.17 (-7.88, 1.53)	
≥65dB	76516	0.99 (0.93, 1.05)		65113	0.99 (0.96, 1.03)		76516	-1.85 (-5.76, 2.06)	
p for trend	540365		0.886	471489		0.952	540365		0.142
**L_{night}**									
<50dB	162260	Reference		142880	Reference		162260	Reference	
50-<55 dB	257045	0.99 (0.95, 1.03)		224864	1.00 (0.97, 1.02)		257045	0.82 (-1.94, 3.58)	
55-<60 dB	40256	1.00 (0.93, 1.08)		34960	1.02 (0.98, 1.06)		40256	-3.20 (-7.92, 1.51)	
60-<65 dB	46994	1.01 (0.94, 1.08)		40344	1.00 (0.96, 1.04)		46994	-2.22 (-6.70, 2.26)	
≥65dB	33810	1.00 (0.92, 1.08)		28441	1.02 (0.97, 1.07)		33810	-8.15 (-13.48, -2.83)	
p for trend	540365		0.869	471489		0.448	540365		0.003
**Adjusted for PM_{10}**									
**$L_{Aeq,16hr}$**									
<55dB	157491	Reference		138696	Reference		157491	Reference	
55-<60 dB	265603	0.98 (0.94, 1.02)		232346	0.99 (0.97, 1.01)		265603	2.62 (-0.14, 5.39)	
60-<65 dB	40755	1.02 (0.95, 1.09)		35334	1.01 (0.97, 1.05)		40755	-3.36 (-8.08, 1.36)	
≥65dB	76516	0.99 (0.93, 1.05)		65113	1.00 (0.97, 1.04)		76516	-2.24 (-6.24, 1.76)	
p for trend	540365		1.000	471489		0.750	540365		0.108
**L_{night}**									
<50dB	162260	Reference		142880	Reference		162260	Reference	
50-<55 dB	257045	0.99 (0.95, 1.03)		224864	1.00 (0.98, 1.02)		257045	0.64 (-2.13, 3.41)	
55-<60 dB	40256	1.00 (0.93, 1.08)		34960	1.03 (0.99, 1.07)		40256	-3.50 (-8.23, 1.23)	
60-<65 dB	46994	1.01 (0.94, 1.08)		40344	1.00 (0.96, 1.04)		46994	-2.60 (-7.12, 1.92)	
≥65dB	33810	1.00 (0.93, 1.09)		28441	1.03 (0.98, 1.08)		33810	-9.05 (-14.51, -3.59)	
p for trend	540365		0.735	471489		0.227	540365		0.001

Exposure	Joint air pollutant-noise model* Term LBW			Joint air pollutant-noise model* Term SGA			Joint air pollutant-noise model* Term birth weight (g)		
	N	OR (95% CI)	p-value	N	OR (95% CI)	p-value	N	MD (95%CI)	p-value
**Adjusted for O_3**									
**$L_{Aeq,16hr}$**									
<55dB	157491	Reference		138696	Reference		157491	Reference	
55-<60 dB	265603	0.98 (0.94, 1.02)		232346	0.99 (0.97, 1.01)		265603	2.31 (-0.44, 5.07)	
60-<65 dB	40755	1.02 (0.95, 1.09)		35334	1.01 (0.97, 1.06)		40755	-4.19 (-8.88, 0.49)	
≥65dB	76516	1.00 (0.94, 1.06)		65113	1.00 (0.97, 1.04)		76516	-4.12 (-7.92, -0.31)	
p for trend	540365		0.865	471489		0.670	540365		0.007
**L_{night}**									
<50dB	162260	Reference		142880	Reference		162260	Reference	
50-<55 dB	257045	0.99 (0.95, 1.03)		224864	1.00 (0.97, 1.02)		257045	0.40 (-2.36, 3.15)	
55-<60 dB	40256	1.01 (0.94, 1.08)		34960	1.03 (0.98, 1.07)		40256	-4.14 (-8.83, 0.56)	
60-<65 dB	46994	1.01 (0.95, 1.08)		40344	1.00 (0.96, 1.04)		46994	-3.64 (-8.07, 0.80)	
≥65dB	33810	1.01 (0.93, 1.09)		28441	1.03 (0.99, 1.08)		33810	-11.09 (-16.25, -5.93)	
p for trend	540365		0.622	471489		0.205	540365		<0.001

*Models are adjusted as follows. Term LBW: sex, maternal age, birth registration type, tobacco expenditure (COA-level), Carstairs quintile (COA-level), individual-level ethnicity, gestational age as linear and quadratic terms, season of birth, year (linear term) and random intercept for MSOA. Term SGA: maternal age, birth registration type, tobacco expenditure (COA-level), Carstairs quintile (COA-level), season of birth, year (linear term) and random intercept for MSOA. Term birth weight: sex, maternal age, birth registration type, tobacco expenditure (COA-level), Carstairs quintile (COA-level), individual-level ethnicity, gestational age as linear and quadratic terms, season of birth, year (linear term) and random intercept for MSOA. All models are additionally adjusted for the air pollutant stated in the table above.

Supplementary Table 10: Adjusted* odds of term LBW and term SGA associated with trimester-specific air pollutant exposures (models adjusted for road traffic noise co-exposure)

(To allow comparison between trimesters for a given pollutant we present analyses per specific µg/m^3 increments, as the IQR varied between pregnancy and trimester-specific averages).

Exposure	Exposure window	Term LBW OR (95% CI)	Term SGA OR (95% CI)
**NO_2 (per 10 µg/m^3)**	**Pregnancy**	1.04 (1.00, 1.07)	1.01 (0.99, 1.03)
	**Trimester 1**	1.01 (0.98, 1.03)	1.01 (1.00, 1.03)
	**Trimester 2**	1.03 (1.00, 1.05)	1.01 (1.00, 1.03)
	**Trimester 3**	1.03 (1.00, 1.05)	1.00 (0.99, 1.02)
**NO_X (per 20 µg/m^3)**	**Pregnancy**	1.03 (1.00, 1.05)	1.01 (1.00, 1.02)
	**Trimester 1**	1.01 (0.99, 1.03)	1.01 (1.00, 1.02)
	**Trimester 2**	1.02 (1.00, 1.04)	1.01 (1.00, 1.02)
	**Trimester 3**	1.02 (1.00, 1.04)	1.00 (0.99, 1.01)
**$PM_{2.5\ traffic\text{-}exhaust}$ (per 1 µg/m^3)**	**Pregnancy**	1.13 (1.03, 1.23)	1.06 (1.01, 1.12)
	**Trimester 1**	1.07 (0.99, 1.15)	1.05 (1.01, 1.10)
	**Trimester 2**	1.11 (1.03, 1.19)	1.05 (1.01, 1.10)
	**Trimester 3**	1.10 (1.02, 1.19)	1.02 (0.98, 1.07)
**$PM_{2.5\ traffic\text{-}non\text{-}exhaust}$ (per 1 µg/m^3)**	**Pregnancy**	1.08 (0.99, 1.18)	1.02 (0.97, 1.08)
	**Trimester 1**	1.03 (0.96, 1.11)	1.03 (0.98, 1.07)
	**Trimester 2**	1.07 (1.00, 1.16)	1.03 (0.98, 1.07)
	**Trimester 3**	1.06 (0.98, 1.14)	1.00 (0.95, 1.04)
**$PM_{2.5}$ (per 5 µg/m^3)**	**Pregnancy**	1.15 (1.02, 1.30)	1.07 (1.00, 1.15)
	**Trimester 1**	1.02 (0.97, 1.07)	1.03 (1.00, 1.06)
	**Trimester 2**	1.01 (0.96, 1.06)	1.01 (0.98, 1.04)
	**Trimester 3**	1.05 (1.00, 1.10)	0.99 (0.97, 1.02)
**PM_{10} (per 10 µg/m^3)**	**Pregnancy**	1.09 (0.96, 1.25)	1.01 (0.94, 1.09)
	**Trimester 1**	1.03 (0.95, 1.11)	1.04 (0.99, 1.08)
	**Trimester 2**	1.01 (0.94, 1.08)	1.00 (0.96, 1.04)
	**Trimester 3**	1.04 (0.98, 1.11)	0.98 (0.94, 1.02)
**O_3 (per 10 µg/m^3)**	**Pregnancy**	0.96 (0.92, 0.99)	0.99 (0.97, 1.01)
	**Trimester 1**	0.99 (0.96, 1.01)	0.99 (0.97, 1.00)
	**Trimester 2**	0.97 (0.95, 0.99)	1.00 (0.98, 1.01)
	**Trimester 3**	0.98 (0.96, 1.01)	1.00 (0.99, 1.02)

*Term low birth weight models adjusted for: sex, maternal age, birth registration type, tobacco expenditure (COA-level), Carstairs quintile (COA-level), individual-level ethnicity, gestational age as linear and quadratic terms, season of birth, year (linear term), random intercept for MSOA and daytime noise ($L_{Aeq,16hr}$). Total N = 540,365, with 13,804 cases of term LBW. Term SGA models adjusted for: maternal age, birth registration type, tobacco expenditure (COA-level), Carstairs quintile (COA-level), season of birth, year (linear term) and random intercept for MSOA. Total N = 471,489, with 44,966 cases of term SGA.

Supplementary Table 11: Joint air-pollutant-noise models: Adjusted mean difference (95% CI) in birth weight (g) associated with air pollutants (per IQR) and noise amongst term births

Exposure	White N	White Term birth weight (g) MD (95%CI)	White p-value	Asian N	Asian Term birth weight (g) MD (95%CI)	Asian p-value	Black N	Black Term birth weight (g) MD (95%CI)	Black p-value
**NO_2 (per IQR)**	286192	-13.02 (-15.91, -10.12)		93555	-5.74 (-11.17, -0.32)		91740	-10.84 (-15.90, -5.78)	
**NO_X (per IQR)**	286192	-13.31 (-16.22, -10.40)		93555	-4.63 (-10.00, 0.73)		91740	-10.01 (-14.96, -5.06)	
**$PM_{2.5\ traffic\text{-}exhaust}$ (per IQR)**	286192	-15.60 (-18.83, -12.37)		93555	-10.06 (-15.74, -4.39)		91740	-8.89 (-14.27, -3.51)	
**$PM_{2.5\ traffic\text{-}non\text{-}exhaust}$ (per IQR)**	286192	-9.93 (-12.48, -7.38)		93555	-2.65 (-7.05, 1.75)		91740	-6.76 (-11.03, -2.49)	
**$PM_{2.5}$ (per IQR)**	286192	-14.66 (-19.64, -9.68)		93555	-6.51 (-15.17, 2.14)		91740	-13.33 (-21.90, -4.77)	
**PM_{10} (per IQR)**	286192	-8.44 (-12.22, -4.65)		93555	-2.08 (-8.60, 4.44)		91740	-5.57 (-12.11, 0.96)	
**O_3 (per IQR)**	286192	5.31 (2.58, 8.05)		93555	5.20 (0.40, 10.01)		91740	5.60 (0.66, 10.55)	
**Adjusted for NO_2**									
**Day-time noise, $L_{Aeq,16hr}$**									
<55dB	87807	Reference		25713	Reference		25181	Reference	
55-<60 dB	140525	1.41 (-2.32, 5.15)		46700	10.96 (4.38, 17.55)		45126	6.59 (-0.20, 13.38)	
60-<65 dB	20879	-4.18 (-10.69, 2.34)		7120	9.49 (-1.67, 20.65)		7335	-2.68 (-14.07, 8.72)	
≥65dB	36981	-1.08 (-6.69, 4.53)		14022	6.06 (-3.33, 15.45)		14098	6.72 (-2.91, 16.35)	
p for trend	286192		0.442	93555		0.212	91740		0.364
**Night-time noise, L_{night}**									
<50dB	90922	Reference		26617	Reference		25343	Reference	
50-<55 dB	135636	-0.49 (-4.23, 3.25)		45157	8.47 (1.90, 15.05)		44076	4.69 (-2.13, 11.51)	
55-<60 dB	20705	-5.56 (-12.08, 0.97)		7064	9.40 (-1.75, 20.56)		7193	0.57 (-10.91, 12.04)	
60-<65 dB	23121	-1.77 (-8.10, 4.56)		8717	9.15 (-1.30, 19.59)		8499	2.29 (-8.62, 13.20)	
≥65dB	15808	-6.31 (-14.20, 1.58)		6000	-9.37 (-22.41, 3.68)		6629	5.48 (-7.37, 18.33)	
p for trend	286192		0.090	93555		0.994	91740		0.581

Models are adjusted as follows: sex, maternal age, birth registration type, tobacco expenditure (COA-level), Carstairs quintile (COA-level), individual-level ethnicity, gestational age as linear and quadratic terms, season of birth, year (linear term) and random intercept for MSOA. All air pollution estimates are adjusted for daytime noise ($L_{Aeq,16hr}$), and noise estimates are adjusted for traffic-related air pollution exposure (NO2). IQR values for air pollutants: NO_2 (per IQR, 8.6 μg/m^3), NO_X (per IQR, 23.7 μg/m^3), $PM_{2.5\ traffic\ exhaust}$ (per IQR, 0.35 μg/m^3), $PM_{2.5\ traffic\ non\text{-}exhaust}$ (per IQR, 0.29 μg/m^3), $PM_{2.5}$ (per IQR, 2.2 μg/m^3), PM_{10} (per IQR, 3.0 μg/m^3), O_3 (per IQR, 8.4 μg/m^3)

Supplementary Appendix 1 - Adjusted joint-exposure generalised additive models (GAMs)

The plots below show smoothing functions with 95% confidence intervals for the association between term birth weight and night-time noise (L_{night}) and air pollutants, in joint-exposure models.

L_{night} is night-time noise. All models are adjusted for sex, maternal age, birth registration type, tobacco expenditure (COA-level), Carstairs quintile (COA-level), individual-level ethnicity, gestational age as linear and quadratic terms, season of birth, and year, in addition to including the air pollutant and/or noise metrics listed.

Model 1: NO_X and L_{night}

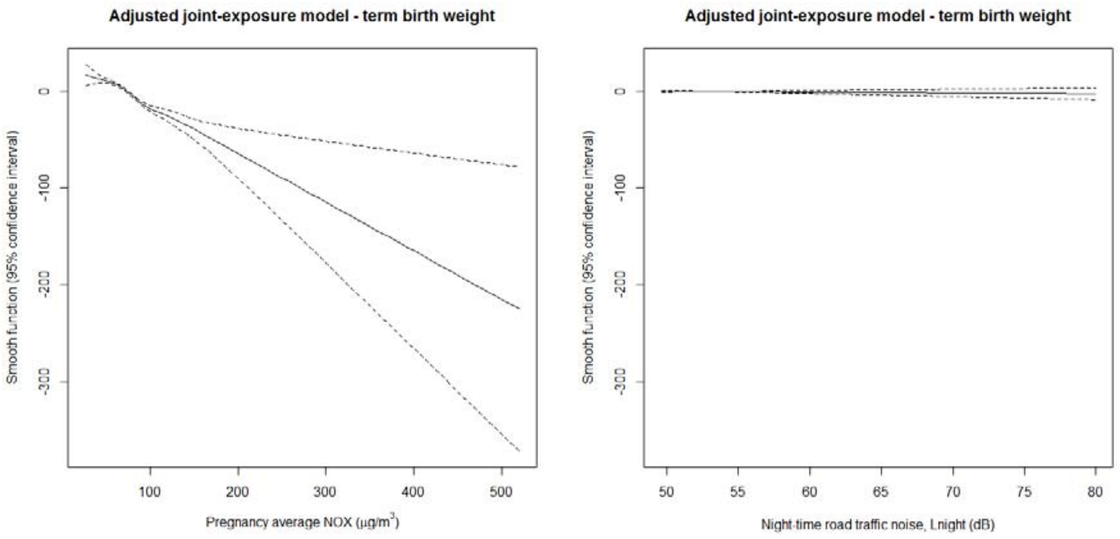

Model 3: $PM_{2.5\ traffic\text{-}exhaust}$ and L_{night}

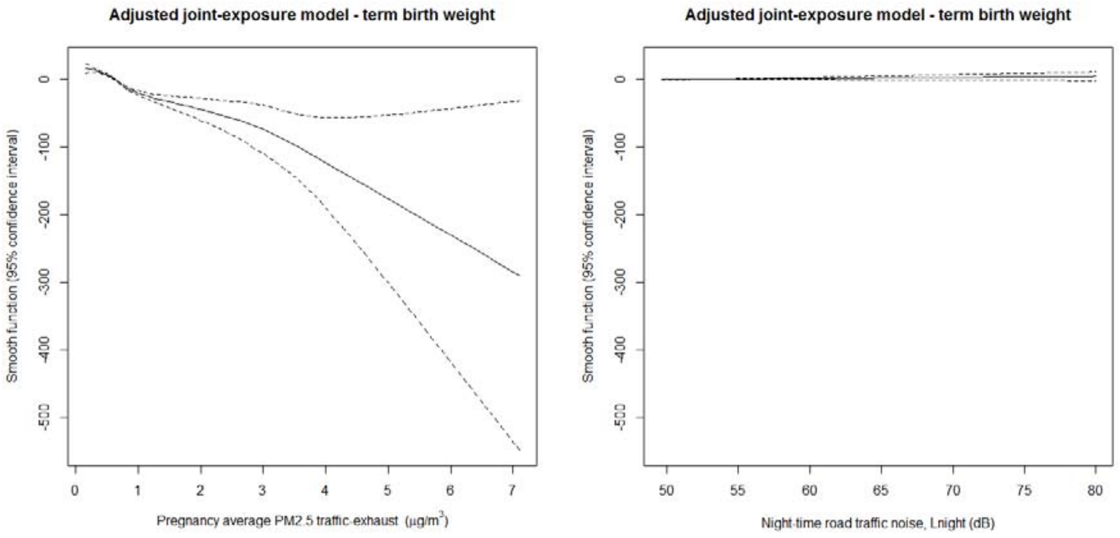

Model 4: $PM_{2.5\ traffic\text{-}non\text{-}exhaust}$ and L_{night}

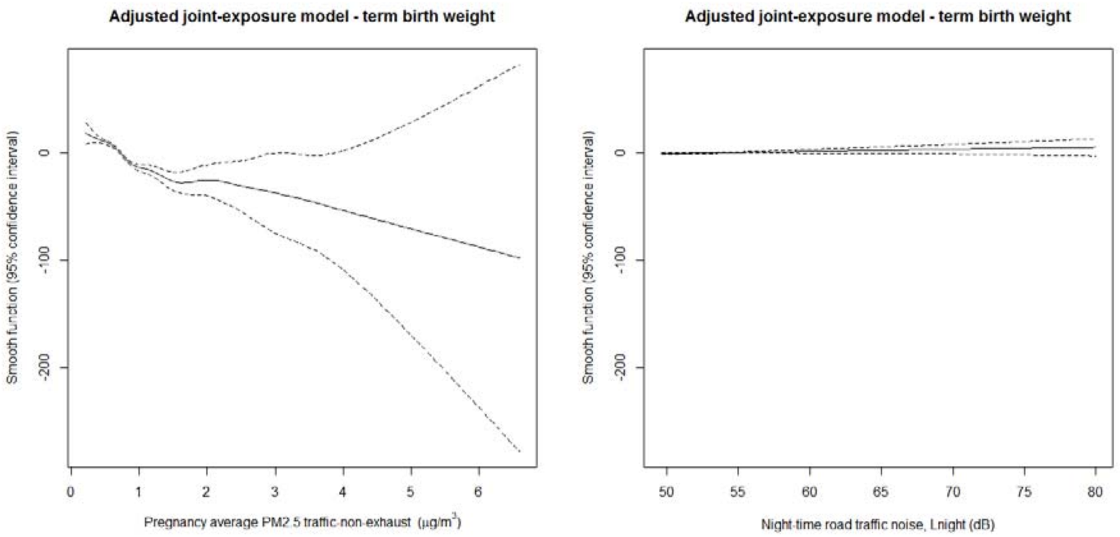

Model 5: $PM_{2.5}$ and L_{night}

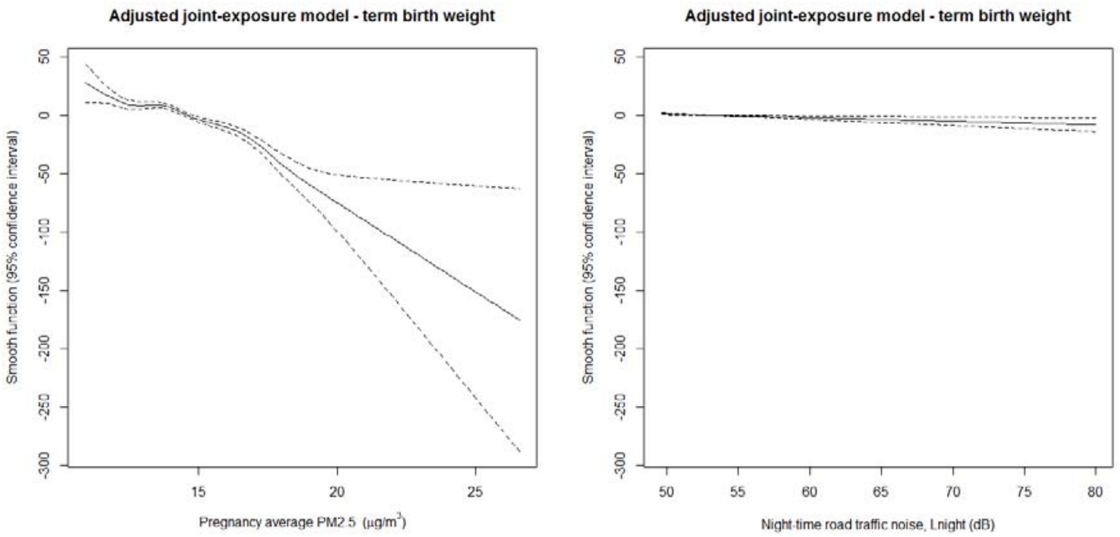

Model 6: PM_{10} and L_{night}

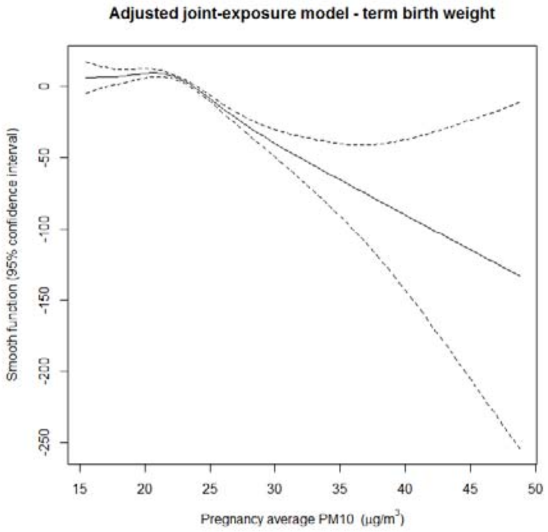

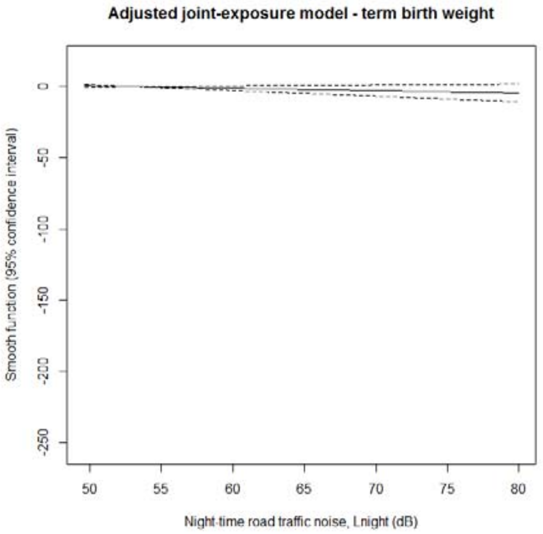

Model 7: O_3 and L_{night}

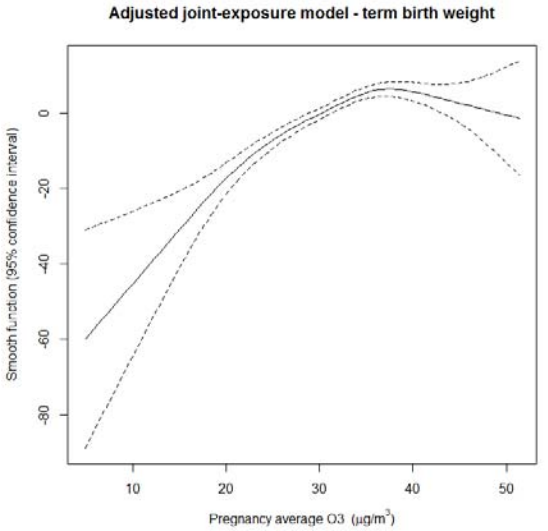

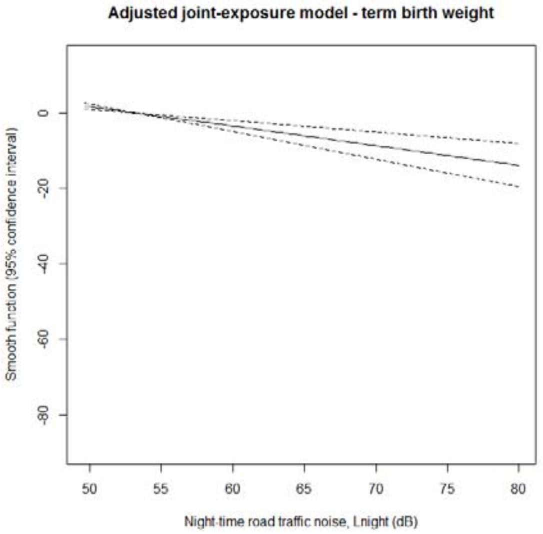

Supplement: Supplementary file 1 — Appendix: Supplementary materials [file smir039236.ww1.pdf]
